# Pre-Clinical Rationale for Amcenestrant Combinations in HER2+/ER+ Breast Cancer

**DOI:** 10.3390/ijms26020460

**Published:** 2025-01-08

**Authors:** Amira F. Mahdi, Niall Ashfield, John Crown, Denis M. Collins

**Affiliations:** 1 Cancer Biotherapeutics Research Group, Life Sciences Institute, School of Biotechnology, Dublin City University, Dublin 9, D09 NR58 Dublin, Ireland; niall.ashfield3@mail.dcu.ie (N.A.); john.crown@ccrt.ie (J.C.); 2 Limerick Digital Cancer Research Centre, Health Research Institute, School of Medicine, University of Limerick, V94 T9PX Limerick, Ireland; 3 Department of Medical Oncology, St. Vincent’s University Hospital, Dublin 4, D04 T6F4 Dublin, Ireland

**Keywords:** breast cancer, oestrogen receptor, HER2, selective oestrogen receptor degrader, amcenestrant

## Abstract

HER2-positive/oestrogen receptor-positive (HER2+/ER+) represents a unique breast cancer subtype. The use of individual HER2- or ER-targeting agents can lead to the acquisition of therapeutic resistance due to compensatory receptor crosstalk. New drug combinations targeting HER2 and ER could improve outcomes for patients with HER2+/ER+ breast cancer. In this study, the pre-clinical rationale is explored for combining amcenestrant (Amc), a selective oestrogen receptor degrader (SERD), with HER2-targeted therapies including trastuzumab, trastuzumab-emtansine (T-DM1) and tyrosine kinase inhibitors (TKIs). The combination of Amc and anti-HER2 therapies was investigated in a panel of four HER2+/ER+ cell lines: BT-474, MDA-MB-361, EFM-192a and a trastuzumab-resistant variant BT-474-T. Proliferation (IC_50_ and matrix combination assays) was determined using acid phosphatase assays. HER2/ER and intracellular signalling pathway protein levels/activity were investigated by western blot. Apoptosis was assessed using caspase 3/7 assays. Additivity and synergy were observed between Amc and the TKIs neratinib, lapatinib and tucatinib in all cell lines. Amc increased the anti-proliferative effect of trastuzumab in MDA-MB-361 and BT-474-T. Addition of Amc also increased anti-proliferative efficacy of T-DM1 in BT-474-T. TKI/Amc combinations reduced p-HER2 and ER levels and resulted in increased apoptosis. Higher ER expression in MDA-MB-361 and BT-474-T was associated with greater potential for synergy. In conclusion, the combination of Amc- and HER2-targeted treatments has potential as a therapeutic strategy for the treatment of HER2+/ER+ breast cancer and warrants further clinical investigation to validate safety and efficacy in patients.

## 1. Introduction

The human epidermal growth factor receptor 2 positive (HER2+) breast cancer subtype makes up 15–20% of breast cancers, with approximately 70% of HER2+ breast cancers also classed as oestrogen receptor α positive (ER+) [1]. There are multiple HER2-targeted therapies approved for the treatment of HER2+ breast cancer, including the monoclonal antibody therapies (mAbs) trastuzumab and pertuzumab, the tyrosine kinase inhibitors (TKIs) lapatinib, tucatinib and neratinib, and the antibody drug conjugates (ADCs) T-DM1 and T-DXd [2]. A number of ER-targeting drugs are approved for the treatment of the ER+ breast cancer subtype including the selective oestrogen receptor modulator (SERM) tamoxifen, the aromatase inhibitor (AI) letrozole, or the selective oestrogen receptor degrader (SERD) fulvestrant [3]. However, HER2+/ER+ breast cancer is primarily treated with HER2-targeted therapies, even though the complex crosstalk and reciprocal signalling between the HER2 and ER pathways can lead to the development of resistance when HER2 or ER therapies are used as single agents [4,5]. The combined therapeutic targeting of HER2 and ER may overcome compensatory HER2/ER resistance mechanisms and has shown benefit both in vitro and in vivo [6,7]. The addition of trastuzumab, a HER2-targeted mAb, to AIs such as letrozole and anastrozole endocrine therapy has shown benefit in several trials, including the eLEcTRA and TAnDEM studies [8,9]. The combination of lapatinib, a reversible EGFR and HER2 TKI, has been investigated in combination with letrozole or fulvestrant with mixed results [10,11,12]. More recently, both the SUMMIT and ExteNET trials have shown the combination of neratinib, an irreversible pan-HER TKI, with endocrine therapies including fulvestrant and physicians’ choice of SERM/AI to have efficacy in HER2+/ER+ breast cancer patients [13,14].

Considering the large repertoire of both HER2 and ER-targeted therapies, the most beneficial combination needs consideration. SERDs are utilised in cases of advanced ER+ breast cancer that have progressed on previous endocrine therapy [15]. Unlike SERMs or AIs that decrease ligand-stimulated ER activity, SERDs bind to ER and trigger its proteasomal degradation, overcoming both ligand-independent signalling and *ESR1* mutations, thus eliciting a more complete abrogation of signalling [16]. Fulvestrant was approved in 2002 for the treatment of ER+ metastatic breast cancer [15]. However, the poor bioavailability and pharmacokinetic profile of fulvestrant requires monthly administration by intramuscular injection, high loading doses and weeks to establish effective steady-state concentrations in patients [17,18,19]. Moreover, the drawbacks of intramuscular injections such as pain and the need to attend clinic can have a negative impact on patient quality of life, therapy adherence and treatment length [17,19]. Thus, over the past decade, there has been a strong focus on finding alternative, orally available SERDs that can improve upon fulvestrant. Dozens of oral SERDS are in early, pre-clinical and clinical investigation, with elacestrant becoming the first oral SERD approved in early 2023 [20], more than 20 years after the approval of fulvestrant.

Amcenestrant (SAR439859) is a non-steroidal, orally available SERD which shows an equivalency to fulvestrant in vitro, and improved efficacy in PDX models [21]. Amcenestrant is a potent antagonist of ER, eliciting 98% degradation efficiency at concentrations as low as 0.2 nM, with no off-target activity [21,22]. Amcenestrant also demonstrated potency in tumours harbouring an *ESR1* mutation that confers resistance to fulvestrant [21]. The phase I/II, first-in-human trial of amcenestrant, AMEERA-1, was conducted in postmenopausal women with pre-treated HER2−/ER+ breast cancer [23]. This trial showed antitumor activity for amcenestrant, irrespective of *ESR1* mutation status, with a clinical benefit rate (complete response + partial response + stable disease ≥  24 weeks) of 28.3% in a heavily pre-treated cohort, and no discontinuation due to treatment emergent adverse events [23,24]. These promising results led to the initiation of amcenestrant monotherapy and combination trials in various HER2−/ER+ patient cohorts. However, the clinical development of amcenestrant was discontinued in 2022 without clinical investigation in HER2+/ER+ breast cancer [25].

Oral SERDs may offer a new approach to combinatorial therapy with HER2-targeted therapies for the treatment of HER2+/ER+ breast cancer. In this rationale-building pre-clinical study, we examine the efficacy of amcenestrant alone and in combination with the HER2-targeting TKIs lapatinib, tucatinib and neratinib, the mAb trastuzumab and the ADC T-DM1 in a panel of HER2+/ER+ breast cancer cell lines.

## 2. Results

### 2.1. Amcenestrant Displays Differential Anti-Proliferative Efficacy Against ER+ Breast Cancer Cell Lines

**Figure 1:**
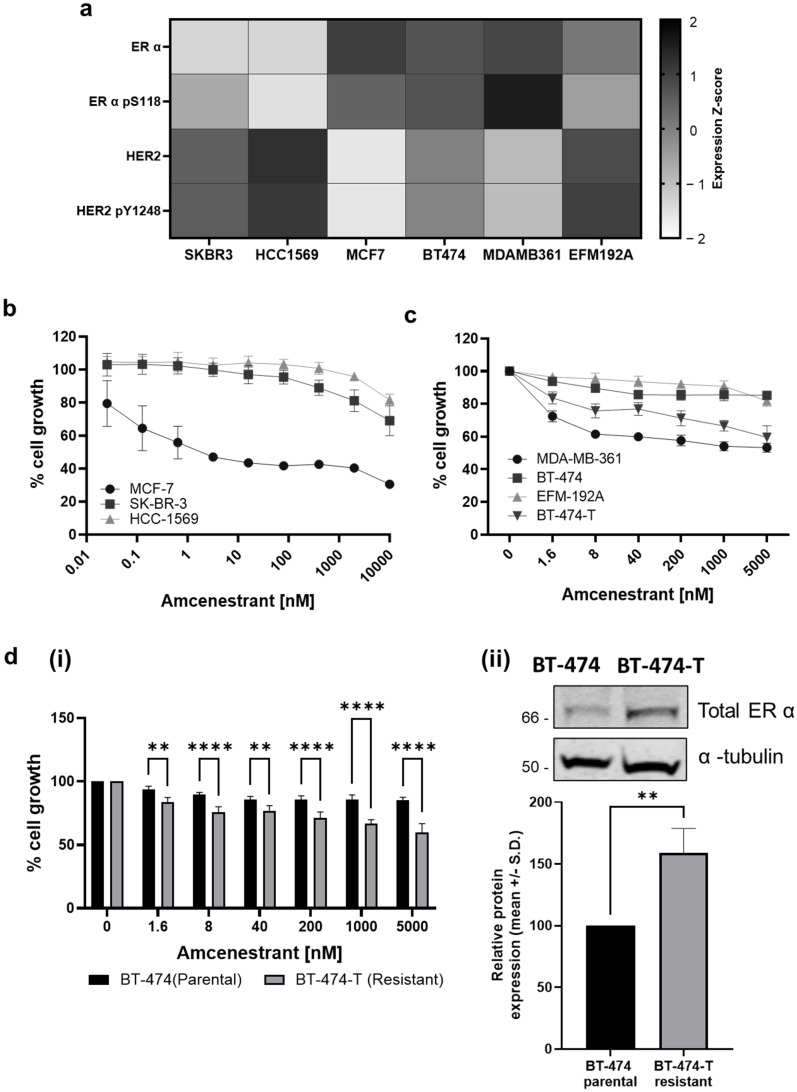
The effect of amcenestrant on breast cancer cell line models. (**a**) RPPA expression data for proteins of interest in the breast cancer cell lines, values displayed are Z-scored expression of Log2(RPPA signal) as exported from CCLE. (**b**) Relative cell growth following amcenestrant treatment in HER2−/ER+ MCF-7 and HER2+/ER− SK-BR-3, HCC-1569 cell lines. Cell proliferation was assessed by acid phosphatase proliferation assay following 5 days of drug exposure. Data points represent average % cell growth ± SD. (**c**) Relative cell growth following amcenestrant treatment in HER2+/ER+ MDA-MB-361, EFM-192A, BT-474 and BT-474-T. Data points represent average % cell growth ± SD. (**d**) (**i**) direct comparison of the % growth between parental BT-474 and the trastuzumab-resistant variant BT-474-T. Data points represent average % cell growth ± SD. Differences were assessed using two-way ANOVA; (**ii**) Densitometry analysis of relative ERα protein expression from three independent experiments, Student’s *t*-test. ** = *p* ≤ 0.01.**** = *p* ≤ 0.0001. n = 3.

Six breast cancer cell lines representing different clinical subtypes (HER2−/ER+, HER2+/ER−, HER2+/ER+) were tested for response to amcenestrant (Table 1). Total and phosphorylated protein levels of HER2 and ERα were assessed in the cell line panel using publicly available RPPA data from the Cancer Cell Line Encyclopaedia (Figure 1a). The three HER2+/ER+ cell lines MDA-MB-361, BT-474 and EFM-192A have variable expression of HER2 and ERα, with the MDA-MB-361 cell line displaying the highest ER/p-ER expression and the lowest HER2 expression, and EFM-192A showing the highest HER2 expression and lowest ERα expression (Figure 1a). The BT-474 cell line exhibited expression levels of ER and HER2 that fell between the other two cell lines. The three HER2+/ER+ cell lines were also subjected to western blotting analysis, which again showed MDA-MB-361 to have the highest level of ERα protein expression, whereas EFM-192A expressed the lowest levels (Supplementary Figure S1).

In proliferation assays (Figure 1b), MCF-7 cells (HER2−/ER+) were sensitive to amcenestrant, with an IC_50_ value of 13.5 ± 4.96 nM. An IC_50_ for amcenestrant was not achieved at 10 µM for the HER2+/ER− cell lines SK-BR-3 and HCC-1569 (Figure 1b). Of the HER2+/ER+ cell lines, amcenestrant had the greatest effect in the MDA-MB-361 line (Figure 1c), reducing relative cell proliferation to 53.3 ± 2.8 % at the highest concentration tested. An IC_50_ for amcenestrant was not achieved in the BT-474 and EFM-192a cell lines (Figure 1c, Supplementary Table S2). Interestingly, a model of acquired trastuzumab resistance, BT-474-T, displayed resistance to trastuzumab, increased proliferation rate (Supplementary Figure S2) and greater sensitivity to amcenestrant than the parental BT-474 (Supplementary Table S2 and Figure 1d(i)). Protein ERα expression was found to be upregulated in BT-474-T compared to the parental BT-474 cell line (Figure 1d(ii)), along with increased levels of activated p-EGFR, p-HER2 and p-ERK1/2 (Supplementary Figure S3).

### 2.2. Amcenestrant Augments the Anti-Proliferative Activity of HER2-Targeting TKIs in HER2+/ER+ Cell Lines

For all HER2+/ER+ cell lines, the HER2-targeting agents neratinib, tucatinib, lapatinib and T-DM1 potently reduced cell growth at nanomolar (or ng/mL) concentrations. Neratinib was the most potent TKI in all cell lines, producing IC_50_ values in the range of 1.1–1.8 nM (Table 2). The IC_50_ point for each compound was compared with or without 1 µM amcenestrant as a quantitative assessment of the shift in growth curve associated with the addition of amcenestrant. For all cell lines tested, the addition of amcenestrant reduced the IC_50_ of the tested HER2 TKIs, with a significant decrease observed in 3/4 cell lines for neratinib and lapatinib, and 4/4 cell lines for tucatinib (Table 2).

Amcenestrant reduced the IC_50_ value for both neratinib and tucatinib in the BT-474 cell line as outlined in Table 2 and illustrated in the fixed ratio combination assays in Supplementary Figure S4. For the MDA-MB-361 cell line, the addition of amcenestrant augmented TKI effects, reducing the IC_50_ values for all TKIs tested (Table 2, Supplementary Figure S5). Interestingly, while amcenestrant lowered the IC_50_ for T-DM1 in BT-474, BT-474-T and EFM-192a, it increased it in the MDA-MB-361 cell line. Significant decreases for tucatinib and lapatinib IC_50_ values in the presence of amcenestrant were observed in the EFM-192A cell line (Table 2, Supplementary Figure S6). For BT-474-T, amcenestrant significantly lowered the IC_50_ of all three TKIs and T-DM1 (Table 2, Supplementary Figure S7). However, due to the limited efficacy of amcenestrant in the cell lines, it was not possible to ascertain synergy/antagonism using fixed-ratio combination analysis. For this reason, we went on to further investigate the combinations using matrix combinations, as outlined in Section 2.4.

### 2.3. Anti-Proliferative Benefit of Trastuzumab and Amcenestrant Combination Is Dependent on Initial Sensitivity to Amcenestrant

**Figure 2:**
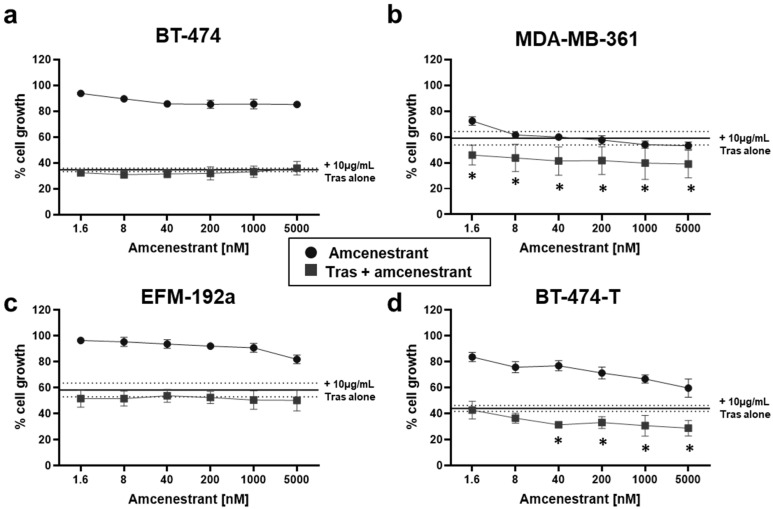
The impact of amcenestrant on the anti-proliferative effects of trastuzumab (Tras) in amcenestrant-responsive cell lines. Cells were treated with 10 µg/mL Tras alone (solid line, dotted lines denote ± SD), varying concentrations of amcenestrant alone (circle), or with a combination of Tras and amcenestrant (square) for 5 days in (**a**) BT-474, (**b**) MDA-MB-361, (**c**) EFM-192a, and (**d**) BT-474-T cell lines. The difference between % cell growth when treated with Tras alone and the Tras/amcenestrant combination was assessed using two-way ANOVA. * indicates *p* > 0.05, n = 3 with error bars denoting ± SD.

The combination of amcenestrant with the standard-of-care HER2-targeting mAb trastuzumab was also assessed. A fixed concentration of 10 µg/mL trastuzumab was combined with a range of amcenestrant combinations, up to 5000 nM. As shown in Figure 2, any further decrease in cell proliferation caused by the addition of amcenestrant to trastuzumab was associated with the cell line’s sensitivity to amcenestrant alone. BT-474 and EFM-192A cells were less responsive to amcenestrant and the addition of trastuzumab did not improve the anti-proliferative effect vs. trastuzumab alone (Figure 2a,c). For the MDA-MB-361 and BT-474-T cell lines, the addition of amcenestrant resulted in a significant decrease in cell growth compared to trastuzumab alone (Figure 2b,d). The BT-474-T cell line displayed a 9.34% reduction in trastuzumab-induced growth inhibition compared to the BT-474 parental cell line (Figure 2a,d). While this change is modest, it is impactful as the BT-474-T cell line grows faster than the parental cell line (Supplementary Figure S3) and displays persistent cell growth when challenged with trastuzumab.

### 2.4. HER2-Targeted Agents and Amcenestrant Are Synergistic in HER2+/ER+ Cell Lines

**Figure 3:**
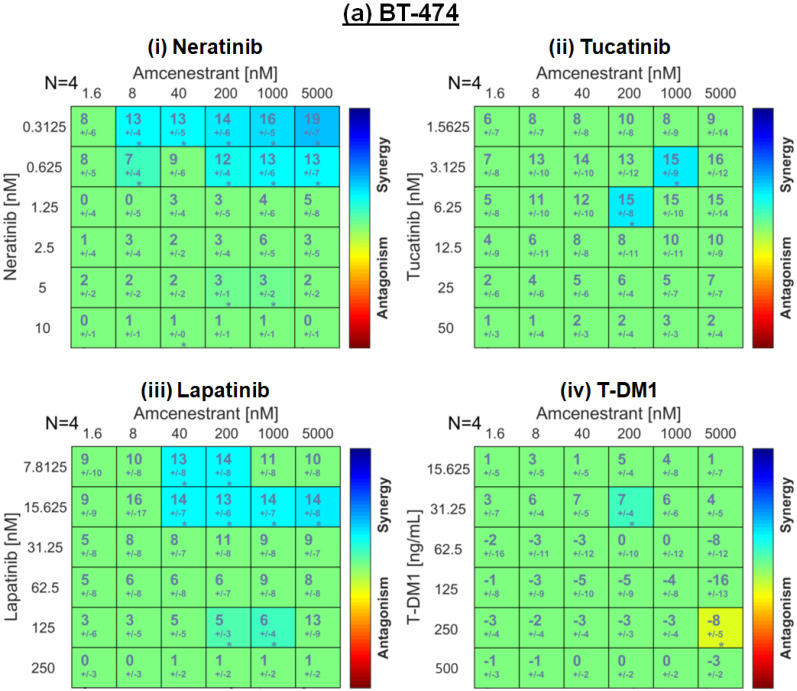

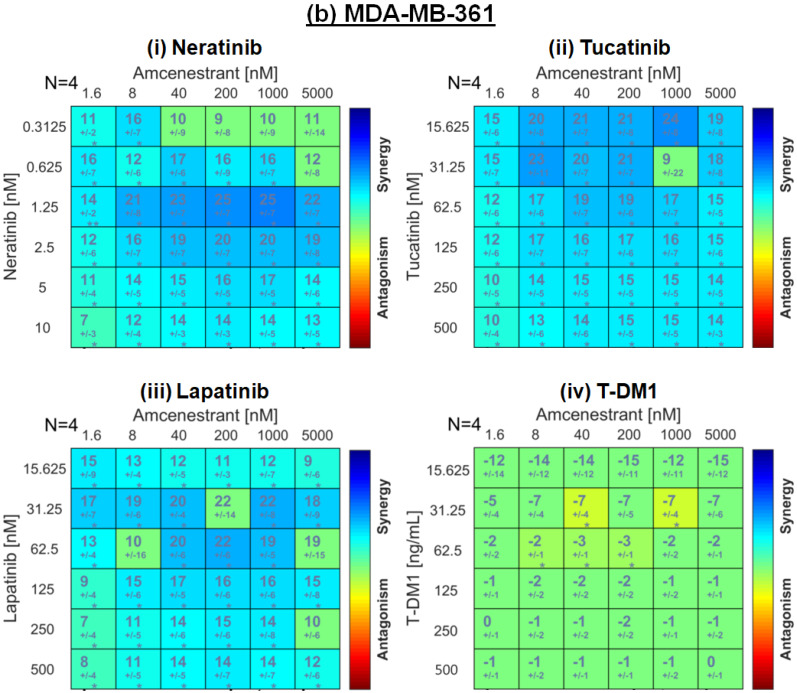

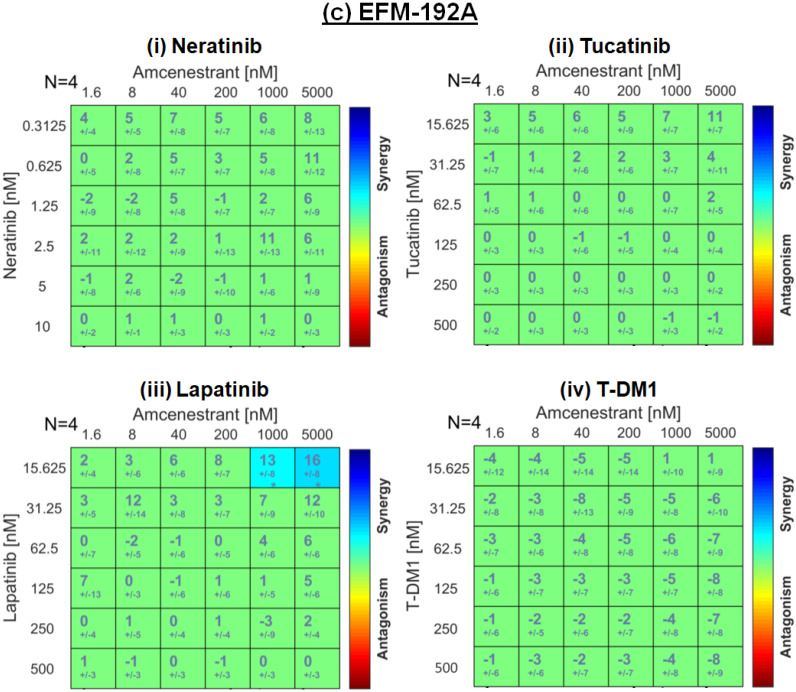

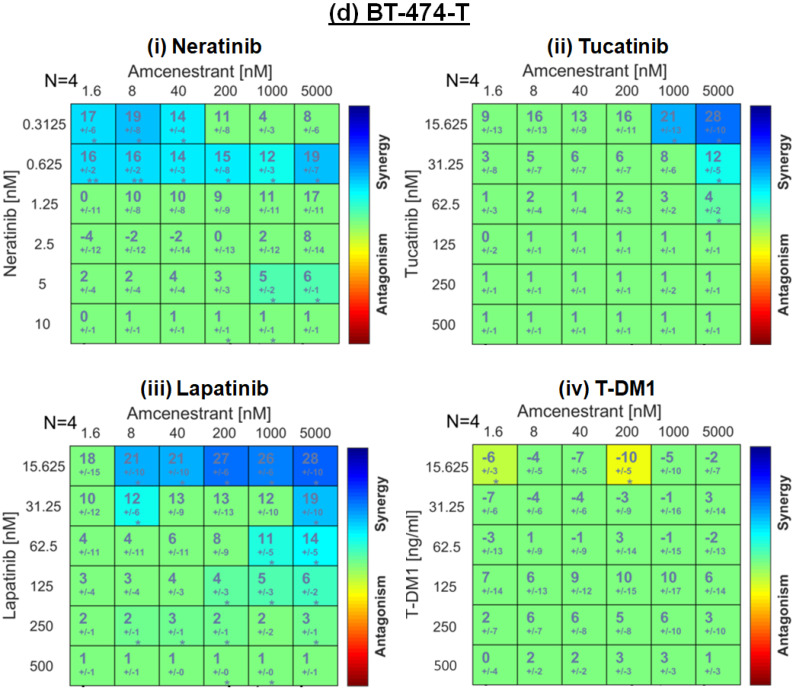
Loewe synergy tables generated from matrix combination assays examining synergy between HER2-targeted agents and amcenestrant in HER2+/ER+ cell lines (**a**) BT-474, (**b**) MDA-MB-361, (**c**) EFM-192a and (**d**) BT-474-T. Amcenestrant (1.6–5000 nM) was combined with (**i**) neratinib (**ii**) tucatinib, (**iii**) lapatinib or (**iv**) T-DM-1. The boxes are coloured according to the synergism/antagonism scale provided with green representing additivity. * indicates *p*  <  0.05 or ** indicates *p*  <  0.001 according to the one-sample t-test in Combenefit, n = 4.

To assess if there was a synergistic, additive or antagonistic relationship between test compounds, matrix assays were carried out (dose–response values depicted in Supplementary Figure S8) and the Loewe synergy score for each drug combination was calculated using Combenefit software (vers 2.021) (Figure 3) [28]. Synergistic combinations are denoted by a Loewe synergy score greater than zero and are indicated in blue, with green indicating an additive effect and yellow–red indicating antagonism.

In MDA-MB-361 cells, TKI concentrations below peak plasma concentrations (neratinib C_max_ ~150 nM [29], tucatinib C_max_ ~1200 nM [30] and lapatinib C_max_ ~3000 nM [31]) synergised with clinically achievable concentrations of amcenestrant (peak plasma, 7200 nM, communicated by Sanofi) (Figure 3b). EFM-192a displayed the least synergy (Figure 3c), followed by BT-474 (Figure 3a). The combination of amcenestrant and T-DM1 was additive in BT-474, MDA-MB-361 and EFM-192a. Higher synergy levels were observed in the trastuzumab-resistant BT-474-T cells compared to their parental cell line, BT-474, (Figure 3d versus Figure 3a). To broadly compare the cell line and drug combinations, the overall data produced by the Combenefit software are displayed in Supplementary Table S3, confirming the highest summative scores in the MDA-MB-361 cell line.

### 2.5. Amcenestrant and HER2-Targeted Agent Combinations Enhance Inhibition of Intracellular Signalling Pathways

**Figure 4:**
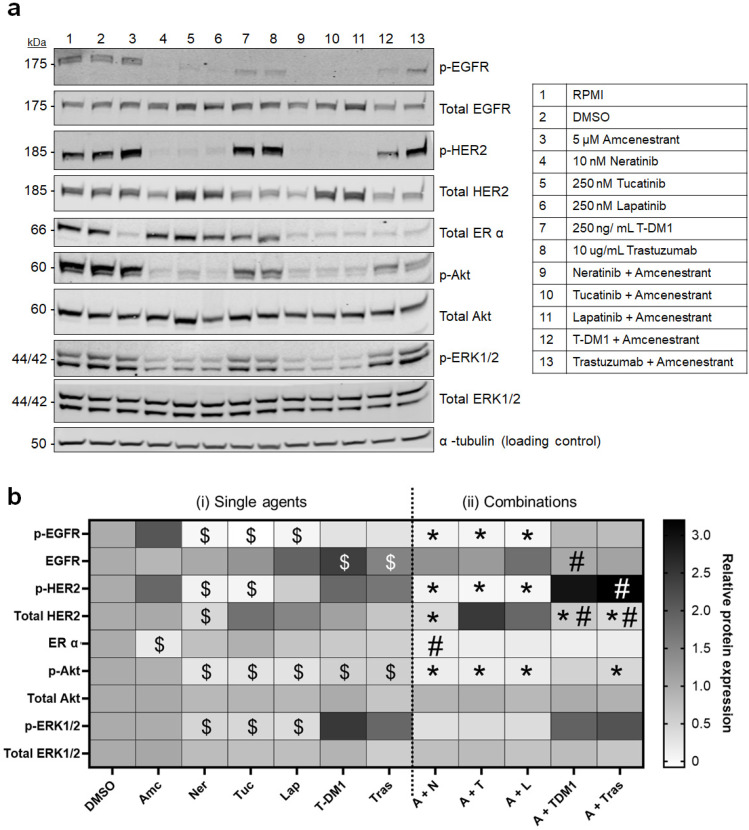
Western blot analysis of drug target and intracellular signalling in MDA-MB-361 following treatment with amcenestrant, HER2-targeted therapies and combinations. (**a**) Western blots for total HER2, p-HER2, total EGFR, p-EGFR, total ERK1/2, p-ERK1/2, Akt and p-Akt after 24 h treatments. α-tubulin was used as a loading control. Blot images are representative of independent experiments carried out in triplicate. (**b**) Heatmap of relative protein expression from three independent experiments. Densitometry for each protein band was normalised to its corresponding α-tubulin loading control for total proteins, and to both α-tubulin and to total protein for phosphorylated proteins. Protein level was expressed as fold-change from DMSO control. $ indicates significantly different from control (DMSO or RPMI) using Student’s *t* test. * indicates significantly different from amcenestrant treatment alone using one way ANOVA, # indicates significantly different from HER2-targeted agent treatment alone, n = 3.

As the MDA-MB-361 cell line displayed the most sensitivity to amcenestrant/HER2-targeted drug combinations (Figure 1c and Figure 3b), an in-depth assessment of drug target activity and intra-cellular signalling was conducted in this cell line by Western blot. MDA-MB-361 cells were treated with single agent HER2-targeted agents and amcenestrant, as well as combinations, for 24 h (Figure 4a). Changes in protein levels were determined by densitometry (Figure 4b). Levels of total Akt and total ERK1/2 remained constant across treatment conditions, but treatments containing HER2 TKIs caused a significant decrease in the phosphorylated forms of both (Figure 4b(i)). Tucatinib and lapatinib displayed an increase in total HER2 and EGFR levels, whereas neratinib triggered a significant decrease compared to DMSO controls, in agreement with previous findings from our lab [32,33]. Phosphorylated HER2 and EGFR levels were reduced by TKI treatments, with stronger inhibition seen in amcenestrant combinations. All six treatments containing amcenestrant showed a potent and significant decrease in the expression of ERα compared to DMSO control (Figure 4b(i)). Overall, TKI-containing treatments elicited stronger inhibition of activated receptors compared to the mAb- and ADC-based treatments, as seen in p-EGFR, p-HER2 and p-ERK1/2 (Figure 4b(i)). Combination treatment lowered expression of p-EGFR, p-HER2 and p-Akt, compared to amcenestrant alone, but not compared to the HER2-targeted agent alone (Figure 4b(ii)). Amcenestrant + neratinib was the only combination to significantly lower expression of ERα compared to neratinib alone. Total HER2 expression was reduced by amcenestrant + T-DM1 and amcenestrant + trastuzumab combinations compared to single agents, but it was accompanied by an increase in the relative proportion of p-HER2 (Figure 4b(ii)).

### 2.6. Combinations of HER2-Targeted Agents and Amcenestrant Increase Apoptosis Induction Compared to Single Agent Treatment in MDA-MB-361 Cells

**Figure 5:**
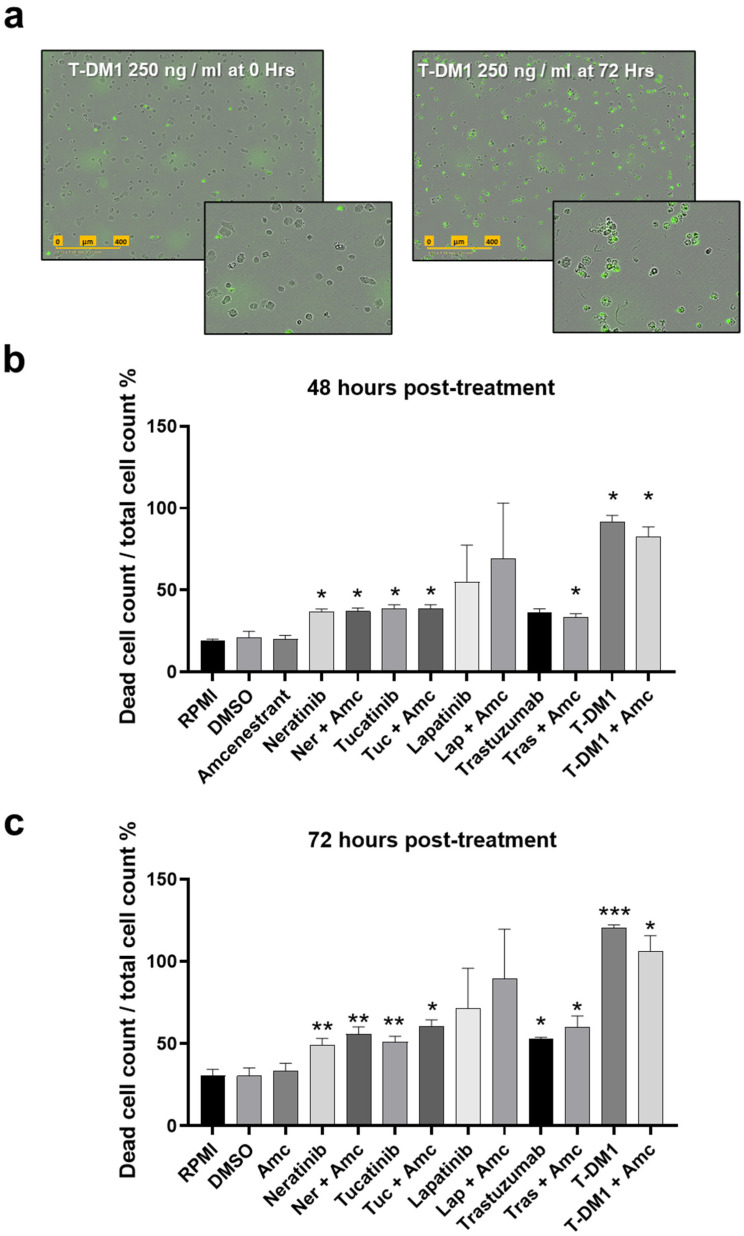
Apoptosis induction in MDA-MB-361 cells after treatment with amcenestrant, HER2-targeted agents and combinations. A total of 5 µM amcenestrant (Amc), 10 nM neratinib (Ner), 250 nM tucatinib (Tuc) and lapatinib (Lap), 250 ng/mL T-DM1 and 10 µg/mL trastuzumab (Tras) were used. (**a**) Representative images of cells at the start of the experiment and following the induction of apoptosis with T-DM1 after 72 h. Apoptotic cells display increased green fluorescence. Scale bar indicates 400 µm. (**b**) Proportion of dead cells to total cell count at 48 h post-treatment timepoint for all treatment conditions, mean ± SEM. (**c**) Proportion of dead cells to total cell count at 72 h post-treatment timepoint for all treatment conditions, mean ± SEM. * indicates significantly different from RPMI control, following one-way ANOVA. * = *p* ≤ 0.05, ** = *p* ≤ 0.01. *** = *p* ≤ 0.001. n = 3.

Apoptosis in the MDA-MB-361 cell lines was assessed through live cell imaging of green fluorescence associated with activated caspase 3/7, illustrated in Figure 5a. Supplementary Figure S9 shows measurements taken every 12 h for all treatments. Concentrations tested are outlined in Figure 4 (5 µM amcenestrant, 10 nM neratinib, 250 nM tucatinib and lapatinib, 250 ng/mL T-DM1, 10 μg/mL Trastuzumab). T-DM1 and T-DM1 + amcenestrant consistently induced the highest levels of apoptosis from 24 h. Statistical comparison of treatments vs. untreated control (RPMI) was conducted for the 48 h and 72 h timepoints (Figure 5b,c). The addition of DMSO did not cause any measurable increase in apoptosis. Single agent TKIs and T-DM1 were more potent than amcenestrant. Single agent neratinib, tucatinib and T-DM1 elicited significant increases in apoptosis at 48 h. For these three treatments, additional amcenestrant also showed increased apoptosis compared to the untreated control, with the further combination of trastuzumab + amcenestrant also reaching significance compared to the untreated control (Figure 5b). At 72 h, single agent neratinib, tucatinib, trastuzumab and T-DM1 and combinations thereof with amcenestrant caused significant increases in cell death compared to the untreated control. T-DM1 elicited a four-fold increase in apoptotic cells at 72 h, with the percentage of apoptotic cells/total cells rising to over 100% as the cells lose normal morphology but the DNA-bound green fluorescently labelled dye persists. The addition of amcenestrant to the HER2-targeted TKIs resulted in numerically higher levels of apoptotic cells compared to respective single agent HER2-targeted therapy at 72 h. However, when single agent HER2 TKIs were directly compared to TKI + amcenestrant, the increases were not statistically significant (Figure 5c), suggesting apoptosis was driven primarily by HER2-targeted agents. Conversely, the addition of amcenestrant appeared to marginally reduce induction of apoptosis by T-DM1 (Figure 5c). Investigation into the combination treatment effect on cell migration using wound healing assays was also conducted (Supplementary Figure S10). While the amcenestrant/lapatinib and amcenestrant/trastuzumab combinations displayed minor increases in wound width, no significant changes were observed as the MDA-MB-361 cell line did not display wound-closing abilities.

## 3. Discussion

There is a growing consensus that the combination of anti-HER2 and endocrine therapies is an underutilized clinical option that may help overcome resistance, de-escalate the use of chemotherapeutics and improve patient survival in HER2+/ER+ breast cancer [6]. A recent meta-analysis of randomised controlled trials in patients with HER2+/ER+ breast cancer found that endocrine therapy-containing regimes elicited better efficacy than chemotherapy-containing regimes [34]. However, the translation of this rationale to patient treatment is limited by a lack of specific clinical trials and limited options for ER-targeting therapeutic compounds.

In this pre-clinical study, it is suggested that the orally available next generation SERD amcenestrant may have potential in combination with HER2-targeted agents for the treatment of HER2+/ER+ breast cancer. We provide new evidence on the pre-clinical efficacy of amcenestrant and synergy in combination with HER2-targeted therapies in HER2+/ER+ breast cancer models.

Initial investigations confirmed amcenestrant has a potent effect on the growth of ER+ cell line MCF-7 (Figure 1b). This is consistent with previous studies on the synthesis and discovery of amcenestrant [21,22]. Figure 1b also shows the lack of efficacy in HER2+/ER- cell lines HCC-1569 and SK-BR-3 demonstrating the selectivity of amcenestrant for cells reliant on ER expression for growth. Despite all being classified as HER2+/ER+, the four cell line models utilised in this study had varying responses to amcenestrant (Figure 1a,c). The sensitivity to amcenestrant appears to correlate with ER expression (Figure 1a,c); the higher the ER expression, the better the response to amcenestrant. This was further highlighted through the BT-474-T model that expressed higher ER levels than the parental BT-474 cell line and was more sensitive to amcenestrant (Figure 1d). This may indicate that ER expression levels or ER signalling dependency in HER2+/ER+ breast cancers could be biomarkers of response to amcenestrant and amcenestrant/HER2-targeted therapy combinations.

The activation of oestrogen signalling following acquisition of resistance to HER2 therapies has been reported previously. Giuliano et al. observed increased ER and downstream target expression in both cell line xenograft and patient samples following treatment with lapatinib and trastuzumab [35]. Several other studies have also shown ER activation to provide a therapeutic escape pathway from lapatinib treatment in vitro [36,37]. An investigation of tucatinib in HER2+/ER+ cell lines also showed the upregulation of ER following tucatinib treatment and found that the combination of fulvestrant and palbociclib (targeting CDK4/6) is required to overcome tucatinib resistance in HER2+/ER+ xenografts [38], with a resultant clinical trial showing promising efficacy of the tucatinib/letrozole/palbociclib combination in patients with HER2+/ER+ metastatic breast cancer [39]. In vitro investigation in HER2+/ER+ models has also shown an increase in ER activation following neratinib treatment, which is abrogated upon addition of fulvestrant [7]. It should also be noted that in the current study, the HER2+/ER+ cell line with the greatest sensitivity to amcenestrant also had the lowest levels of HER2 (Figure 1d). The ratio of HER2 to ER may be another potential biomarker of response to amcenestrant and amcenestrant combinations.

Our initial combination experiments demonstrated that amcenestrant augmented the anti-proliferative effects of neratinib, tucatinib and lapatinib (Table 2). Furthermore, the pre-clinical data in this study suggest that amcenestrant will add value to trastuzumab treatment only when the tumour is sensitive to single agent amcenestrant (Figure 2). This lends further emphasis to identifying biomarkers of response to amcenestrant with clinical utility. We also found the dual targeting of both HER2 and ERα elicited synergistic or additive results in HER2+/ER+ cell line models (Figure 3). All three TKIs tested, neratinib (pan-HER family inhibitor), tucatinib (HER2-specific inhibitor) [40] and lapatinib (dual HER2/EGFR inhibitor) [41], displayed additivity or synergy in all cell lines tested, with particularly strong synergy evident in the MDA-MB-361 cell line (Figure 3b). It is possible synergy was associated with greater inhibition of signalling pathways (for example, the numerically lower levels of p-HER2 observed in the TKI/amcenestrant combination treatments compared to single agents (Figure 4)) leading to higher levels of apoptosis (Figure 5). However, in our analysis, apoptosis appears primarily driven by the HER2-targeting agents. It can be hypothesised that the cell cycle arrest effects, well-evidenced for SERDs such as fulvestrant [42,43], may also be contributing to the synergy between anti-HER2 agents and amcenestrant. The more complete inhibition of HER family signalling provided by irreversible, pan-HER TKIs like neratinib may make such compounds more efficacious than reversible inhibitors like lapatinib and tucatinib or mAbs like trastuzumab [41] by reducing intra-HER family and inter-HER/ER family compensatory resistance mechanisms. This is supported by findings from Wang et al., who demonstrated that resistance to lapatinib-containing regimens activates ER signalling, and that a more complete blockade of HER2 is needed by combining lapatinib and trastuzumab, along with endocrine therapy, to provide the greatest regression in HER2+/ER+ xenograft models [44]. Arpino et al. reported similar outcomes when combining trastuzumab and pertuzumab, and the EGFR targeting TKI gefitinib, and endocrine therapy in HER2+/ER+ xenograft tumours [45].

The potential for synergy between neratinib and fulvestrant has also been demonstrated, in both HER2+/ER+ and HER2-mutated/ER+ in vitro models [7,46] and in the ExteNET clinical trial [13]. In this trial, 93% of HER2+/ER+ patients who received neratinib for one year after trastuzumab-based adjuvant therapy also received concomitant endocrine therapy. This extended dual-targeting of the HER and ER pathways resulted in an improvement in invasive disease-free survival at 5-years follow up (hazard ratio = 0.6, 95% CI 0.43–0.83) [47]. Intriguingly, when a less complete HER family blockade is used in the adjuvant setting, such as trastuzumab in the HERA trial, HER2+ patients with higher ER expression (relative to HER2 fluorescence in situ hybridization (FISH)) derived significantly less benefit in both disease-free survival and overall survival [48]. Amcenestrant offers comparable activity to fulvestrant with regards to ER degradation and transcriptional inhibition, without the limitations associated with fulvestrant such as poor bioavailability and the necessity of intramuscular injection [22,49]. The study by Shomali et al. [21] conducted an extensive comparison of amcenestrant compared to the standard-of-care SERD fulvestrant. The study concluded amcenestrant has a similar in vitro profile to fulvestrant, in terms of ER target gene downregulation and inhibition of cell growth. Amcenestrant also performed better than fulvestrant when measuring tumour regression in a PDX model of ER+ breast cancer [21].

The combination of T-DM1 and amcenestrant has an additive or antagonistic effect in most of the cell lines tested in this study (Figure 3). The basis of this reduced efficacy of T-DM1 compared to TKIs was not investigated further in this manuscript. It is well established that HER2 TKI treatment with lapatinib can increase ERα expression and activity in vitro [37,44,50], but less is known regarding the ability of ADCs to mediate resistance by doing likewise. The lack of synergy observed between T-DM1 and amcenestrant could be attributed to the maytansine payload, as chemotherapeutics are traditionally considered antagonistic with endocrine therapy [6]. The addition of amcenestrant to T-DM1 in the BT-474-T resistant cell line unexpectedly re-sensitised the cell line to T-DM1, reducing the IC_50_ by nearly 3-fold. Further investigation of the role of ER in T-DM1 resistance mechanisms in this model is required [51]. The recent WSG-ADAPT-TP (NCT01779206) trial showed the combination of T-DM1 and endocrine therapy to be effective and allowed for the omission of standard chemotherapy without compromising 5-year invasive disease-free survival [52], suggesting further investigation of oral SERDs plus ADC combinations are warranted in trastuzumab-refractory HER2+/ER+ breast cancer.

In 2022, the development program for amcenestrant was discontinued after the AMEERA-5 trial (amcenestrant + palbociclib vs. letrozole + palbociclib [53]) and the AMEERA-3 trial (amcenestrant vs. standard endocrine monotherapy [54]) did not show a benefit for amcenestrant in HER2−/ER+ breast cancers. Amcenestrant has not therefore been investigated clinically in HER2+/ER+ breast cancer. Our pre-clinical findings have highlighted the potential utility of amcenestrant in HER2+/ER+ breast cancer and suggest that HER2-targeted therapy/amcenestrant combinations should be further interrogated in this indication. Based on our results, further in vivo investigations are warranted to provide additional pre-clinical rationale for clinical studies. Amcenestrant has successfully passed Phase I trials demonstrating tolerance in humans [24], and the tested TKIs in this study are already approved for use beyond the first line setting. Thus, there is a strong rationale that a TKI/amcenestrant combination would be tolerable and potentially efficacious in a cohort of HER2+/ER+ breast cancer patients who may have received several therapy lines.

## 4. Materials and Methods

### 4.1. Cell Lines and Reagents

MCF-7 (RRID:CVCL_0031), SK-BR-3 (RRID:CVCL_0033), HCC-1569 (RRID:CVCL_1255), MDA-MB-361 (RRID:CVCL_0620), EFM-192a (RRID:CVCL_1812) and BT-474 (RRID:CVCL_0179) cell lines were obtained from the American Type Culture Collection (ATCC). All cell lines were cultured in RPMI-1640 supplemented with 10% foetal bovine serum (FBS, Merck, Dublin, Ireland), and maintained at 37 °C in a humidified, 5% CO_2_ incubator. Generation of the acquired trastuzumab-resistant cell lines BT474-T has been described previously [55]. Drug-resistant cell lines were maintained in the presence of 10 μg/mL trastuzumab, until one week before experiments when the drug was withdrawn. Verification of resistance was established using time-lapse microscopy and analysis of cell confluence using Incucyte S3 Live Cell Analysis Instrument (Sartorius, Göttingen, Germany) (Supplementary Figure S2). Cell lines underwent routine testing for *Mycoplasma* and cell lines were authenticated by STR profiling (IDEXX Bioanalytics). Stock solutions of trastuzumab and T-DM1 were obtained from St Vincent’s University Hospital, Dublin, Ireland. Drug stocks (10 mM) were prepared in DMSO and working dilutions were made up in RPMI + 10% FBS. Amcenestrant was provided by Sanofi Inc (Cambridge, CA, USA). All other drugs were sourced from SelleckChem (Cologne, Germany).

### 4.2. Proliferation Assays

Cells were plated at 3 × 10^3^ cells/well for SK-BR-3, 2.5 × 10^4^ cells/well for HCC-1569 and 5 × 10^3^ cells/well for remaining cell lines in 100 µL media in 96-well plates and incubated for 24 h before drug treatment. Cells were incubated in the presence of test drugs for 5 days. Drug was then removed, cells washed with PBS and 100 µL para-nitrophenol phosphate substrate in 0.1 M sodium acetate buffer with 0.1% Triton X-100 was added to each well. Plates were incubated for 1–2 h at 37 °C before 50 µL of 1 M sodium hydroxide was added to each well and the resulting absorbance read at 450 nm (Biotek Synergy HT microplate reader, Agilent Technologies, Inc., Santa Clara, CA, USA). Growth of treated cells was expressed as a % of untreated control. DMSO controls were included on each plate. For fixed ratio drug combinations, cells were treated as outlined in Supplementary Table S1. For matrix combination assays, 1:5 (amcenestrant) or 1:2 (TKIs/T-DM1) serial dilutions were used.

### 4.3. Cell Lysates and Western Blotting

Cells were plated at 5 × 10^5^ cells/10 mL in 10 cm cell culture-treated petri dishes and left to adhere overnight. Cells were treated with drug for 24 h before being placed on ice, washed with PBS, and lysed via scraping with RIPA buffer containing protease inhibitor cocktail, sodium orthovanadate and PMSF (all Sigma Aldrich, Dublin, Ireland). Lysates were centrifuged to remove insoluble cell pellets, quantified using a BCA Protein Assay kit (Thermo Fisher Scientific, Dublin, Ireland). A total of 20 μg of protein was separated by SDS-PAGE using 4–12% Bis-Tris polyacrylamide gels (Invitrogen), then transferred to nitrocellulose membranes using the iBlot transfer system (Invitrogen, Waltham, MA, USA). Membranes were blocked in NET wash buffer (1.5 M NaCl, 0.05 M EDTA, 0.5 M Tris, 0.5% Triton X-100, 10% *w/v* gelatine) before being incubated with primary antibodies at 4 °C overnight and secondary antibodies for 1 h at room temperature. The primary antibodies used were HER2 (Calbiochem #OP15), p-HER2 Y1221/1222 (CST #2249), EGFR (CST #2232), p-EGFR Y1173 (CST #4407), ERK1/2 (CST #4695), p-ERK1/2 T202/Y204 (CST #9101), Akt (CST #9272), p-Akt S473 (CST #9271), ERα (Invitrogen, #MA1-39540) and α-tubulin (Sigma #T5168). Secondary antibodies IR Dye800 and IR Dye 680 in mouse and rabbit (Licor, Cambridge, UK) were used for detection. Blots were visualized and the densitometry carried out using the Licor Odyssey scanner and the Image Studio software version 3.0 (RRID:SCR_015795). All densitometry signals were normalised to α-tubulin expression. Uncropped western blot images are available in File S1.

### 4.4. Apoptosis Assays

Cells were seeded in 96-well plates at a density of 5 × 10^3^ cells per well, in 100 µL media and incubated for 24 h. Cells were treated with drugs diluted in media containing IncuCyte Caspase 3/7 green dye kit (#4440, Sartorius, Göttingen, Germany) at a final concentration of 1:1000. Final drug concentrations added to the plate were: amcenestrant 5 µM, neratinib 10 nM, tucatinib 250 nM, lapatinib 250 nM, T-DM1 250 ng/mL and trastuzumab 10 µg/mL. Treated plates were imaged every 6 h over 3 days at 10× magnification using phase and green channels and two images per well, using the Incucyte S3 Live Cell Analysis Instrument (Sartorius, Göttingen, Germany). Cell growth and caspase 3/7 activation was analysed using IncuCyte software (Vers. 2022A) basic analyser. The green object count per phase object count was measured to generate the proportion of apoptotic cells within the total cell population.

### 4.5. Scratch Wound Assay

Cells were seeded in 96-well plates at a high density of 15 × 10^3^ cells per well, in 100 µL media and incubated for 24 h, to achieve full confluency. Uniform scratches in the cell monolayer were made using the Incucyte Wound Maker (Sartorius, Göttingen, Germany). Wells were washed with PBS to remove loose cells and media was replaced with drugged media, in concentrations described in Section 4.4. Wells were imaged every 6 h and closure was measured as % area relative the original measured wound. Images were obtained using the Incucyte S3 Live Cell Analysis Instrument (Sartorius, Göttingen, Germany) and analysed using IncuCyte software (Vers. 2022A) wound closure tool.

### 4.6. Statistical Analysis

All results are representative of at least three biological replicates, with points representing mean ± SD unless otherwise stated. Analysis and statistical testing were carried out using Graphpad Prism Vers. 9 for Windows, GraphPad Software, Boston, MA, USA, (www.graphpad.com) (RRID:SCR_002798). Statistical significance was assessed using unpaired, two-way Student’s *t* test for 2 groups. More than 2 groups were compared with one-way or two-way ANOVA. Significance is described by *p*-values, wherein: * = *p* ≤ 0.05, ** = *p* ≤ 0.01. *** = *p* ≤ 0.001 and **** = *p* ≤ 0.0001. Matrix assays were analysed using Combenefit software (Loewe synergy, vers 2.021) [28]. Incucyte assays were analysed using Incucyte software (Vers. 2022A, https://www.sartorius.com/en/products/live-cell-imaging-analysis/live-cell-analysis-software (accessed on 26 December 2024)).

## 5. Conclusions

In conclusion, our pre-clinical results support the combination of amcenestrant and HER2-targeted agents as a therapeutic strategy in HER2+/ER+ breast cancer, particularly if accompanied by further investigation of appropriate biomarkers of response to amcenestrant associated with ER expression and/or dependency.

## Figures and Tables

**Table 1:** Characteristics of the six commercially available breast cancer cell lines used in this study [26,27].

Cell Line Name	Receptor Expression	Source	Disease Type	Molecular Subtype
MCF-7	HER2−/ER+	Metastasis	Adenocarcinoma	Luminal A
SK-BR-3	HER2+/ER−	Metastasis	Adenocarcinoma	HER2-enriched
HCC-1569	HER2+/ER−	Primary	Metaplastic Carcinoma	HER2-enriched
BT-474	HER2+/ER+	Primary	Ductal Carcinoma	Luminal B
MDA-MB-361	HER2+/ER+	Metastasis	Adenocarcinoma	Luminal B
EFM-192A	HER2+/ER+	Metastasis	Adenocarcinoma	Luminal B

**Table 2:** Summary of the IC_50_ values for each HER2-targeted drug in BT-474, MDA-MB-361, EFM-192a and BT-474-T, in the presence or absence of 1 µM amcenestrant. * = *p* ≤ 0.05. ** = *p* ≤ 0.01. *** = *p* ≤ 0.001 based on Student’s *t* test, ns = not significant, SD = standard deviation. n = 3.

**BT-474**			
IC_50_ ± SD			*t* test
	HER2-targeted drug	+1 µM amcenestrant	
*Neratinib* (nM)	1.1 ± 0.04	0.78 ± 0.06	**
*Tucatinib* (nM)	67.01 ± 7.7	35.75 ± 10.6	*
*Lapatinib* (nM)	59.8 ± 8.7	39.3 ± 7.6	ns
*T-DM1* (ng/mL)	98.8 ± 5.3	472.5 ± 16.8	ns
**MDA-MB-361**			
IC_50_ ± SD			*t* test
	HER2-targeted drug	+1 µM amcenestrant	
*Neratinib* (nM)	1.6 ± 0.4	0.45 ± 0.08	*
*Tucatinib* (nM)	18.6 ± 8.1	1.3 ± 0.6	*
*Lapatinib* (nM)	73.2 ± 13.4	12.5 ± 4.4	**
*T-DM1* (ng/mL)	0.06 ± 0.01	0.70 ± 0.23	*
**EFM-192A**			
IC_50_ ± SD			*t* test
	HER2-targeted drug	+1 µM amcenestrant	
*Neratinib* (nM)	1.83 ± 0.2	1.6 ± 0.2	ns
*Tucatinib* (nM)	29.6 ± 2.8	21.7 ± 1.1	*
*Lapatinib* (nM)	70.4 ± 3.3	54.9 ± 3.1	**
*T-DM1* (ng/mL)	0.10 ± 0.04	0.03 ± 0.02	ns
**BT-474-T**			
IC_50_ ± SD			*t* test
	HER2-targeted drug	+1 µM amcenestrant	
*Neratinib* (nM)	1.7 ± 0.2	0.8 ± 0.1	**
*Tucatinib* (nM)	8.6 ± 1.5	2.9 ± 0.5	**
*Lapatinib* (nM)	20.4 ± 0.6	10.9 ± 1.2	***
*T-DM1* (ng/mL)	200.54 ± 14.2	73.46 ± 30.9	**

## Data Availability

The datasets used and/or analyzed during the current study are available from the corresponding author on reasonable request.

## Supplementary Materials

The following supporting information can be downloaded at: https://www.mdpi.com/article/10.3390/ijms26020460/s1.

## References

[1] Arciero C.A., Guo Y., Jiang R., Behera M., O’Regan R., Peng L., Li X. (2019). ER(+)/HER2(+) Breast Cancer Has Different Metastatic Patterns and Better Survival Than ER(-)/HER2(+) Breast Cancer. Clin. Breast Cancer.

[2] Swain S.M., Shastry M., Hamilton E. (2023). Targeting HER2-positive breast cancer: Advances and future directions. Nat. Rev. Drug Discov..

[3] D’Amico P., Cristofanilli M. (2022). Standard of Care in Hormone Receptor-Positive Metastatic Breast Cancer: Can We Improve the Current Regimens or Develop Better Selection Tools?. JCO Oncol. Pract..

[4] Nayar U., Cohen O., Kapstad C., Cuoco M.S., Waks A.G., Wander S.A., Painter C., Freeman S., Persky N.S., Marini L. (2019). Acquired HER2 mutations in ER(+) metastatic breast cancer confer resistance to estrogen receptor-directed therapies. Nat. Genet..

[5] Razavi P., Chang M.T., Xu G., Bandlamudi C., Ross D.S., Vasan N., Cai Y., Bielski C.M., Donoghue M.T.A., Jonsson P. (2018). The Genomic Landscape of Endocrine-Resistant Advanced Breast Cancers. Cancer Cell.

[6] Pegram M., Jackisch C., Johnston S.R.D. (2023). Estrogen/HER2 receptor crosstalk in breast cancer: Combination therapies to improve outcomes for patients with hormone receptor-positive/HER2-positive breast cancer. NPJ Breast Cancer.

[7] Sudhan D.R., Schwarz L.J., Guerrero-Zotano A., Formisano L., Nixon M.J., Croessmann S., Gonzalez Ericsson P.I., Sanders M., Balko J.M., Avogadri-Connors F. (2019). Extended Adjuvant Therapy with Neratinib Plus Fulvestrant Blocks ER/HER2 Crosstalk and Maintains Complete Responses of ER(+)/HER2(+) Breast Cancers: Implications to the ExteNET Trial. Clin. Cancer Res..

[8] Huober J., Fasching P.A., Barsoum M., Petruzelka L., Wallwiener D., Thomssen C., Reimer T., Paepke S., Azim H.A., Ragosch V. (2012). Higher efficacy of letrozole in combination with trastuzumab compared to letrozole monotherapy as first-line treatment in patients with HER2-positive, hormone-receptor-positive metastatic breast cancer-results of the eLEcTRA trial. Breast.

[9] Kaufman B., Mackey J.R., Clemens M.R., Bapsy P.P., Vaid A., Wardley A., Tjulandin S., Jahn M., Lehle M., Feyereislova A. (2009). Trastuzumab plus anastrozole versus anastrozole alone for the treatment of postmenopausal women with human epidermal growth factor receptor 2-positive, hormone receptor-positive metastatic breast cancer: Results from the randomized phase III TAnDEM study. J. Clin. Oncol..

[10] Johnston S., Pippen J., Pivot X., Lichinitser M., Sadeghi S., Dieras V., Gomez H.L., Romieu G., Manikhas A., Kennedy M.J. (2009). Lapatinib combined with letrozole versus letrozole and placebo as first-line therapy for postmenopausal hormone receptor-positive metastatic breast cancer. J. Clin. Oncol..

[11] Guarneri V., Generali D.G., Frassoldati A., Artioli F., Boni C., Cavanna L., Tagliafico E., Maiorana A., Bottini A., Cagossi K. (2014). Double-blind, placebo-controlled, multicenter, randomized, phase IIb neoadjuvant study of letrozole-lapatinib in postmenopausal hormone receptor-positive, human epidermal growth factor receptor 2-negative, operable breast cancer. J. Clin. Oncol..

[12] Burstein H.J., Cirrincione C.T., Barry W.T., Chew H.K., Tolaney S.M., Lake D.E., Ma C., Blackwell K.L., Winer E.P., Hudis C.A. (2014). Endocrine therapy with or without inhibition of epidermal growth factor receptor and human epidermal growth factor receptor 2: A randomized, double-blind, placebo-controlled phase III trial of fulvestrant with or without lapatinib for postmenopausal women with hormone receptor-positive advanced breast cancer-CALGB 40302 (Alliance). J. Clin. Oncol..

[13] Chan A., Moy B., Mansi J., Ejlertsen B., Holmes F.A., Chia S., Iwata H., Gnant M., Loibl S., Barrios C.H. (2021). Final Efficacy Results of Neratinib in HER2-positive Hormone Receptor-positive Early-stage Breast Cancer from the Phase III ExteNET Trial. Clin. Breast Cancer.

[14] Smyth L.M., Saura C., Piha-Paul S.A., Lu J., Mayer I.A., Brufksy A.M., Spanggaard I., Arnedos M., Cutler R.E., Hyman D.M. (2019). Update on the phase II SUMMIT trial: Neratinib + fulvestrant for HER2-mutant, HR-positive, metastatic breast cancer. Ann. Oncol..

[15] Bross P.F., Cohen M.H., Williams G.A., Pazdur R. (2002). FDA drug approval summaries: Fulvestrant. Oncologist.

[16] Lei J.T., Shao J., Zhang J., Iglesia M., Chan D.W., Cao J., Anurag M., Singh P., He X., Kosaka Y. (2018). Functional Annotation of ESR1 Gene Fusions in Estrogen Receptor-Positive Breast Cancer. Cell Rep..

[17] Wang G. (2020). Fulvestrant as a reference antiestrogen and estrogen receptor (ER) degrader in preclinical studies: Treatment dosage, efficacy, and implications on development of new ER-targeting agents. Transl. Cancer Res..

[18] Robertson J.F., Lindemann J., Garnett S., Anderson E., Nicholson R.I., Kuter I., Gee J.M. (2014). A good drug made better: The fulvestrant dose-response story. Clin. Breast Cancer.

[19] Chen Y.C., Yu J., Metcalfe C., De Bruyn T., Gelzleichter T., Malhi V., Perez-Moreno P.D., Wang X. (2022). Latest generation estrogen receptor degraders for the treatment of hormone receptor-positive breast cancer. Expert. Opin. Investig. Drugs.

[20] Hoy S.M. (2023). Elacestrant: First Approval. Drugs.

[21] Shomali M., Cheng J., Sun F., Koundinya M., Guo Z., Hebert A.T., McManus J., Levit M.N., Hoffmann D., Courjaud A. (2021). SAR439859, a Novel Selective Estrogen Receptor Degrader (SERD), Demonstrates Effective and Broad Antitumor Activity in Wild-Type and Mutant ER-Positive Breast Cancer Models. Mol. Cancer Ther..

[22] El-Ahmad Y., Tabart M., Halley F., Certal V., Thompson F., Filoche-Romme B., Gruss-Leleu F., Muller C., Brollo M., Fabien L. (2020). Discovery of 6-(2,4-Dichlorophenyl)-5-[4-[(3S)-1-(3-fluoropropyl)pyrrolidin-3-yl]oxyphenyl]-8,9-dihydro-7H-benzo[7]annulene-2-carboxylic acid (SAR439859), a Potent and Selective Estrogen Receptor Degrader (SERD) for the Treatment of Estrogen-Receptor-Positive Breast Cancer. J. Med. Chem..

[23] Chandarlapaty S., Linden H.M., Neven P., Petrakova K., Bardia A., Kabos P., Braga S.A.D.S., Boni V., Gosselin A., Cartot-Cotton S. (2021). AMEERA-1: Phase 1/2 study of amcenestrant (SAR439859), an oral selective estrogen receptor (ER) degrader (SERD), with palbociclib (palbo) in postmenopausal women with ER+/ human epidermal growth factor receptor 2-negative (HER2-) metastatic breast cancer (mBC). J. Clin. Oncol..

[24] Bardia A., Chandarlapaty S., Linden H.M., Ulaner G.A., Gosselin A., Cartot-Cotton S., Cohen P., Doroumian S., Paux G., Celanovic M. (2022). AMEERA-1 phase 1/2 study of amcenestrant, SAR439859, in postmenopausal women with ER-positive/HER2-negative advanced breast cancer. Nat. Commun..

[25] Press Release: Sanofi Provides Update on Amcenestrant Clinical Development Program. https://www.sanofi.com/en/media-room/press-releases/2022/2022-08-17-05-30-00-2499668.

[26] Ghandi M., Huang F.W., Jane-Valbuena J., Kryukov G.V., Lo C.C., McDonald E.R., Barretina J., Gelfand E.T., Bielski C.M., Li H. (2019). Next-generation characterization of the Cancer Cell Line Encyclopedia. Nature.

[27] Dai X., Cheng H., Bai Z., Li J. (2017). Breast Cancer Cell Line Classification and Its Relevance with Breast Tumor Subtyping. J. Cancer.

[28] Di Veroli G.Y., Fornari C., Wang D., Mollard S., Bramhall J.L., Richards F.M., Jodrell D.I. (2016). Combenefit: An interactive platform for the analysis and visualization of drug combinations. Bioinformatics.

[29] Ito Y., Suenaga M., Hatake K., Takahashi S., Yokoyama M., Onozawa Y., Yamazaki K., Hironaka S., Hashigami K., Hasegawa H. (2012). Safety, efficacy and pharmacokinetics of neratinib (HKI-272) in Japanese patients with advanced solid tumors: A Phase 1 dose-escalation study. Jpn. J. Clin. Oncol..

[30] Metzger Filho O., Leone J.P., Li T., Tan-Wasielewski Z., Trippa L., Barry W.T., Younger J., Lawler E., Walker L., Freedman R.A. (2020). Phase I dose-escalation trial of tucatinib in combination with trastuzumab in patients with HER2-positive breast cancer brain metastases. Ann. Oncol..

[31] Midgley R.S., Kerr D.J., Flaherty K.T., Stevenson J.P., Pratap S.E., Koch K.M., Smith D.A., Versola M., Fleming R.A., Ward C. (2007). A phase I and pharmacokinetic study of lapatinib in combination with infusional 5-fluorouracil, leucovorin and irinotecan. Ann. Oncol..

[32] Collins D.M., Madden S.F., Gaynor N., AlSultan D., Le Gal M., Eustace A.J., Gately K.A., Hughes C., Davies A.M., Mahgoub T. (2021). Effects of HER Family-targeting Tyrosine Kinase Inhibitors on Antibody-dependent Cell-mediated Cytotoxicity in HER2-expressing Breast Cancer. Clin. Cancer Res..

[33] Castel M.E., Conlon N.T., Walsh N., Diala I., Eli L., Crown J., Collins D.M. (2022). Abstract P2-13-36: Comparative time course analysis of the effects of neratinib, lapatinib and tucatinib in an in vitro HER2+ breast cancer model. Cancer Res..

[34] Wang Y., Xu H., Han Y., Wu Y., Sa Q., Wang J. (2023). Identifying the optimal therapeutics for patients with hormone receptor-positive, HER2-positive advanced breast cancer: A systematic review and network meta-analysis. ESMO Open.

[35] Giuliano M., Hu H., Wang Y.C., Fu X., Nardone A., Herrera S., Mao S., Contreras A., Gutierrez C., Wang T. (2015). Upregulation of ER Signaling as an Adaptive Mechanism of Cell Survival in HER2-Positive Breast Tumors Treated with Anti-HER2 Therapy. Clin. Cancer Res..

[36] Liu L., Greger J., Shi H., Liu Y., Greshock J., Annan R., Halsey W., Sathe G.M., Martin A.M., Gilmer T.M. (2009). Novel mechanism of lapatinib resistance in HER2-positive breast tumor cells: Activation of AXL. Cancer Res..

[37] Xia W., Bacus S., Hegde P., Husain I., Strum J., Liu L., Paulazzo G., Lyass L., Trusk P., Hill J. (2006). A model of acquired autoresistance to a potent ErbB2 tyrosine kinase inhibitor and a therapeutic strategy to prevent its onset in breast cancer. Proc. Natl. Acad. Sci. USA.

[38] Shagisultanova E., Crump L.S., Borakove M., Hall J.K., Rasti A.R., Harrison B.A., Kabos P., Lyons T.R., Borges V.F. (2022). Triple Targeting of Breast Tumors Driven by Hormonal Receptors and HER2. Mol. Cancer Ther..

[39] Shagisultanova E., Gradishar W., Brown-Glaberman U., Chalasani P., Brenner A.J., Stopeck A., Parris H., Gao D., McSpadden T., Mayordomo J. (2023). Safety and Efficacy of Tucatinib, Letrozole, and Palbociclib in Patients with Previously Treated HR+/HER2+ Breast Cancer. Clin. Cancer Res..

[40] Collins D.M., Conlon N.T., Kannan S., Verma C.S., Eli L.D., Lalani A.S., Crown J. (2019). Preclinical Characteristics of the Irreversible Pan-HER Kinase Inhibitor Neratinib Compared with Lapatinib: Implications for the Treatment of HER2-Positive and HER2-Mutated Breast Cancer. Cancers.

[41] Conlon N.T., Kooijman J.J., van Gerwen S.J.C., Mulder W.R., Zaman G.J.R., Diala I., Eli L.D., Lalani A.S., Crown J., Collins D.M. (2021). Comparative analysis of drug response and gene profiling of HER2-targeted tyrosine kinase inhibitors. Br. J. Cancer.

[42] Kaminska K., Akrap N., Staaf J., Alves C.L., Ehinger A., Ebbesson A., Hedenfalk I., Beumers L., Veerla S., Harbst K. (2021). Distinct mechanisms of resistance to fulvestrant treatment dictate level of ER independence and selective response to CDK inhibitors in metastatic breast cancer. Breast Cancer Res..

[43] Dolfi S.C., Jager A.V., Medina D.J., Haffty B.G., Yang J.M., Hirshfield K.M. (2014). Fulvestrant treatment alters MDM2 protein turnover and sensitivity of human breast carcinoma cells to chemotherapeutic drugs. Cancer Lett..

[44] Wang Y.C., Morrison G., Gillihan R., Guo J., Ward R.M., Fu X., Botero M.F., Healy N.A., Hilsenbeck S.G., Phillips G.L. (2011). Different mechanisms for resistance to trastuzumab versus lapatinib in HER2-positive breast cancers--role of estrogen receptor and HER2 reactivation. Breast Cancer Res..

[45] Arpino G., Gutierrez C., Weiss H., Rimawi M., Massarweh S., Bharwani L., De Placido S., Osborne C.K., Schiff R. (2007). Treatment of human epidermal growth factor receptor 2-overexpressing breast cancer xenografts with multiagent HER-targeted therapy. J. Natl. Cancer Inst..

[46] Croessmann S., Formisano L., Kinch L.N., Gonzalez-Ericsson P.I., Sudhan D.R., Nagy R.J., Mathew A., Bernicker E.H., Cristofanilli M., He J. (2019). Combined Blockade of Activating ERBB2 Mutations and ER Results in Synthetic Lethality of ER+/HER2 Mutant Breast Cancer. Clin. Cancer Res..

[47] Martin M., Holmes F.A., Ejlertsen B., Delaloge S., Moy B., Iwata H., von Minckwitz G., Chia S.K.L., Mansi J., Barrios C.H. (2017). Neratinib after trastuzumab-based adjuvant therapy in HER2-positive breast cancer (ExteNET): 5-year analysis of a randomised, double-blind, placebo-controlled, phase 3 trial. Lancet Oncol..

[48] Loi S., Dafni U., Karlis D., Polydoropoulou V., Young B.M., Willis S., Long B., de Azambuja E., Sotiriou C., Viale G. (2016). Effects of Estrogen Receptor and Human Epidermal Growth Factor Receptor-2 Levels on the Efficacy of Trastuzumab: A Secondary Analysis of the HERA Trial. JAMA Oncol..

[49] Besret L., d’Heilly S., Aubert C., Bluet G., Gruss-Leleu F., Le-Gall F., Caron A., Andrieu L., Vincent S., Shomali M. (2020). Translational strategy using multiple nuclear imaging biomarkers to evaluate target engagement and early therapeutic efficacy of SAR439859, a novel selective estrogen receptor degrader. EJNMMI Res..

[50] Montaser R.Z., Coley H.M. (2018). Crosstalk between ERalpha and Receptor Tyrosine Kinase Signalling and Implications for the Development of Anti-Endocrine Resistance. Cancers.

[51] Hunter F.W., Barker H.R., Lipert B., Rothe F., Gebhart G., Piccart-Gebhart M.J., Sotiriou C., Jamieson S.M.F. (2020). Mechanisms of resistance to trastuzumab emtansine (T-DM1) in HER2-positive breast cancer. Br. J. Cancer.

[52] Harbeck N., Nitz U.A., Christgen M., Kummel S., Braun M., Schumacher C., Potenberg J., Tio J., Aktas B., Forstbauer H. (2023). De-Escalated Neoadjuvant Trastuzumab-Emtansine With or Without Endocrine Therapy Versus Trastuzumab With Endocrine Therapy in HR+/HER2+ Early Breast Cancer: 5-Year Survival in the WSG-ADAPT-TP Trial. J. Clin. Oncol..

[53] Bardia A., Cortes J., Hurvitz S.A., Delaloge S., Iwata H., Shao Z.M., Kanagavel D., Cohen P., Liu Q., Cartot-Cotton S. (2022). AMEERA-5: A randomized, double-blind phase 3 study of amcenestrant plus palbociclib versus letrozole plus palbociclib for previously untreated ER+/HER2- advanced breast cancer. Ther. Adv. Med. Oncol..

[54] Tolaney S.M., Chan A., Petrakova K., Delaloge S., Campone M., Iwata H., Peddi P.F., Kaufman P.A., De Kermadec E., Liu Q. (2023). AMEERA-3: Randomized Phase II Study of Amcenestrant (Oral Selective Estrogen Receptor Degrader) Versus Standard Endocrine Monotherapy in Estrogen Receptor-Positive, Human Epidermal Growth Factor Receptor 2-Negative Advanced Breast Cancer. J. Clin. Oncol..

[55] Canonici A., Gijsen M., Mullooly M., Bennett R., Bouguern N., Pedersen K., O’Brien N.A., Roxanis I., Li J.L., Bridge E. (2013). Neratinib overcomes trastuzumab resistance in HER2 amplified breast cancer. Oncotarget.

