# Supplementary material for: Pre-Clinical Rationale for Amcenestrant Combinations in HER2+/ER+ Breast Cancer
Source: Int J Mol Sci. 2025 Jan 8;26(2):460. doi: 10.3390/ijms26020460 (PMC11765389; doi:10.3390/ijms26020460)
Supplement: Supplementary file 1 [file ijms-26-00460-s001.zip › 18.12.2024 IJMS Amcenestrant Blots post review.pdf]

Supplementary Material

Title: Pre-Clinical Rationale for Amcenestrant Combinations in HER2+/ER+ Breast Cancer

Author list: Amira F. Mahdi [1 2]*, Niall Ashfield [1], John Crown [1 3], Denis M. Collins [1] *

Affiliations:
1: Cancer Biotherapeutics Research Group, Life Sciences Institute, School of Biotechnology, Dublin City University, Dublin 9, D09 NR58 Dublin, Ireland; niall.ashfield3@mail.dcu.ie (N.A.); john.crown@ccrt.ie (J.C.)
2: Limerick Digital Cancer Research Centre, Health Research Institute, School of Medicine, University of Limerick, V94 T9PX Limerick, Ireland
3: Department of Medical Oncology, St. Vincent's University Hospital, Dublin 4, D04 T6F4 Dublin, Ireland

* Correspondence: amira.mahdi@ul.ie (A.F.M.); denis.collins@dcu.ie (D.M.C.)

Original Images for Blots

Original, uncropped blot images, as exported from LiCor Odyssey Sc infrared scanner. All blots were incubated with Licor Secondary antibodies IR Dye800 or IR Dye 680 in mouse or rabbit. Blots were visualized and the densitometry carried out using the Licor Odyssey scanner and the Image Studio software version 3.0 (RRID:SCR_015795). All densitometry signals were normalised to α-tubulin expression

Original blot images For Figure 1 D	
ER α	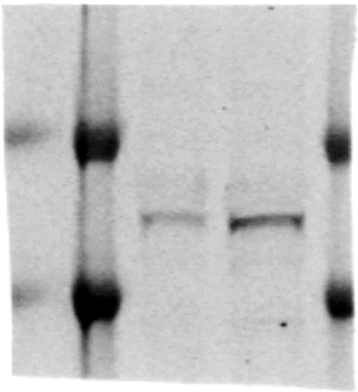
α tubulin	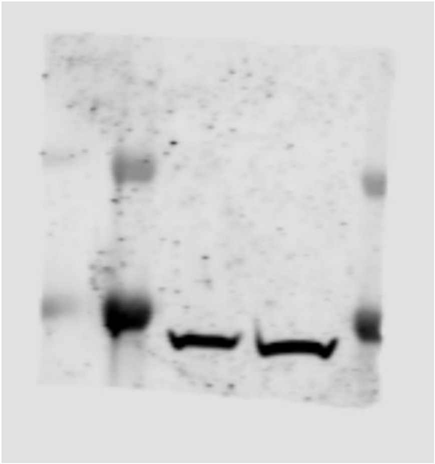

Original blot images For Figure 4 A	
p-EGFR	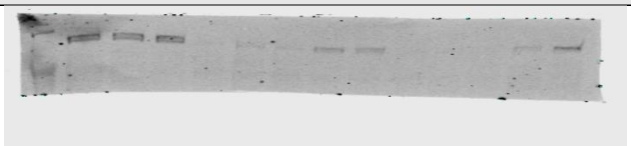
Total EGFR	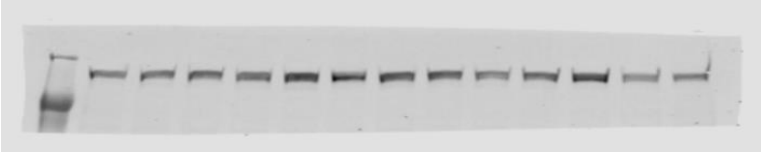
p-HER2	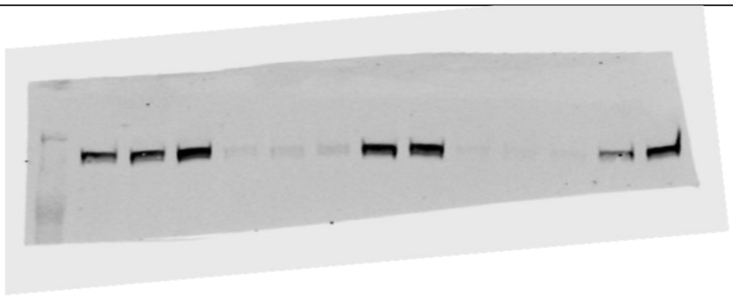

Total HER2	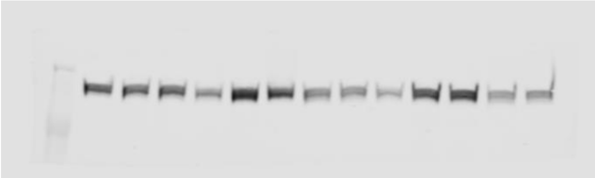
ER α	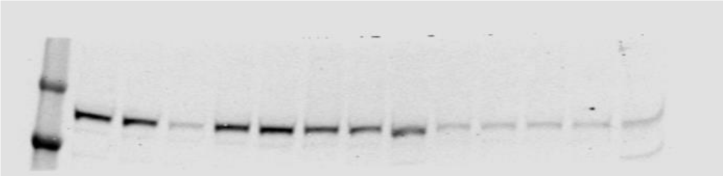
p-Akt	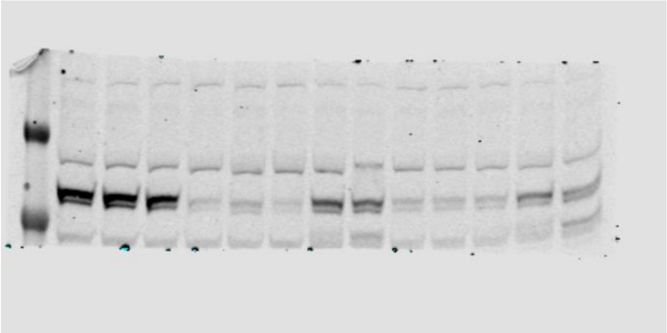
Total Akt	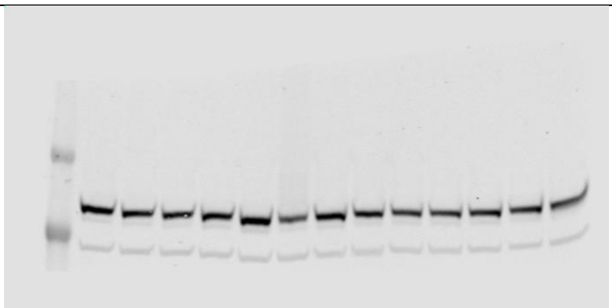
p-ERK1/2	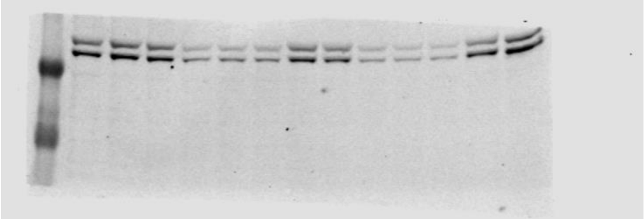
Total ERK1/2	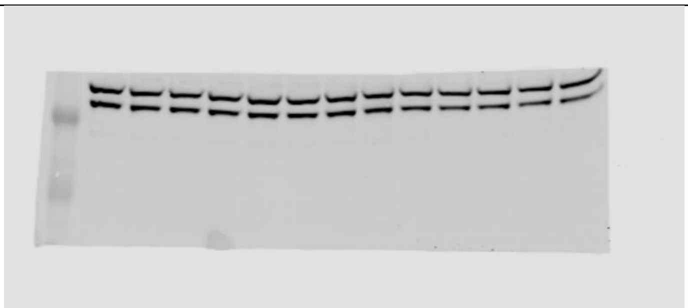

α tubulin	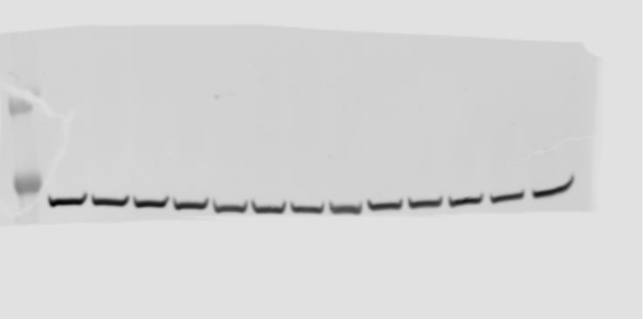

Original blot images For Supplemental Figure S1	
p-EGFR	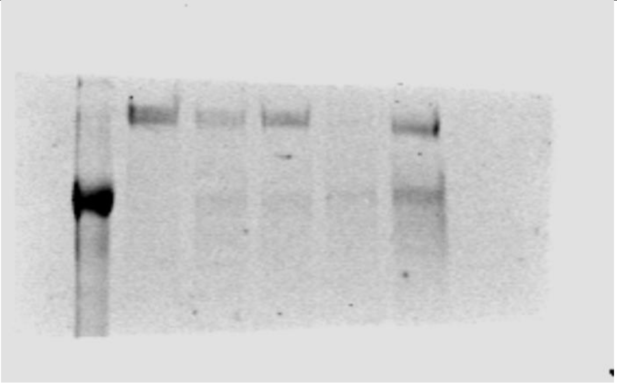
Total EGFR	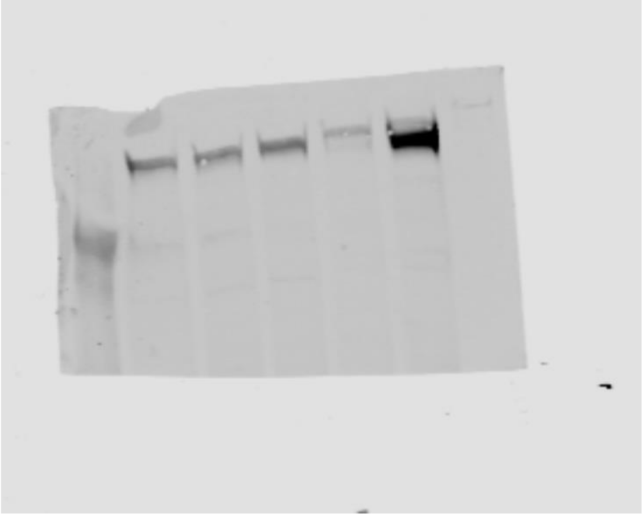
p-HER2	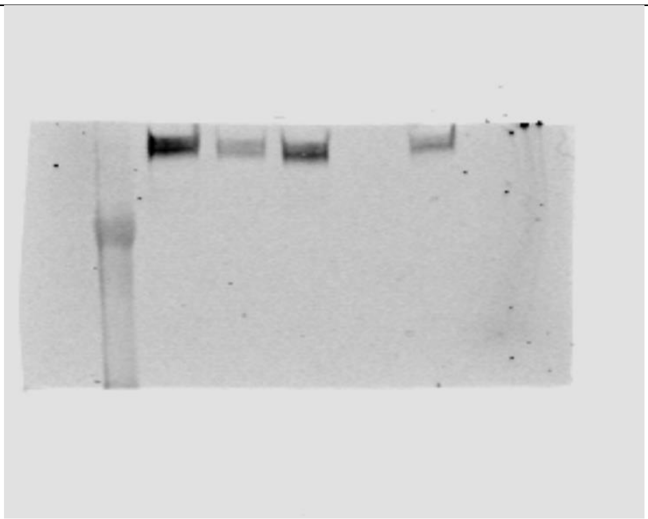

Total HER2	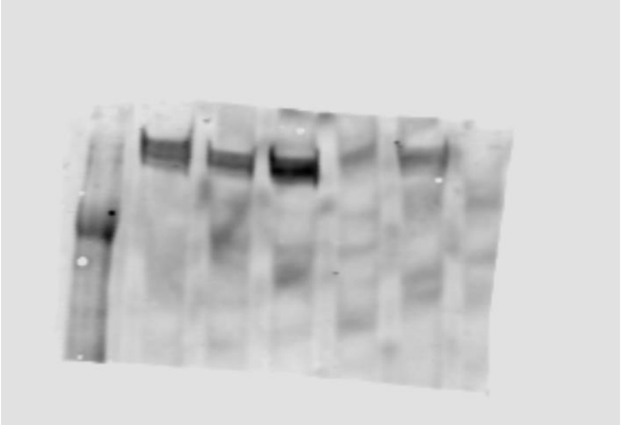
Total ER	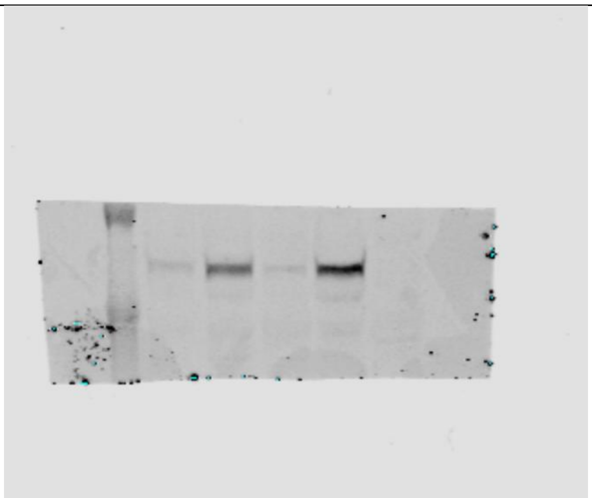
p-AKT	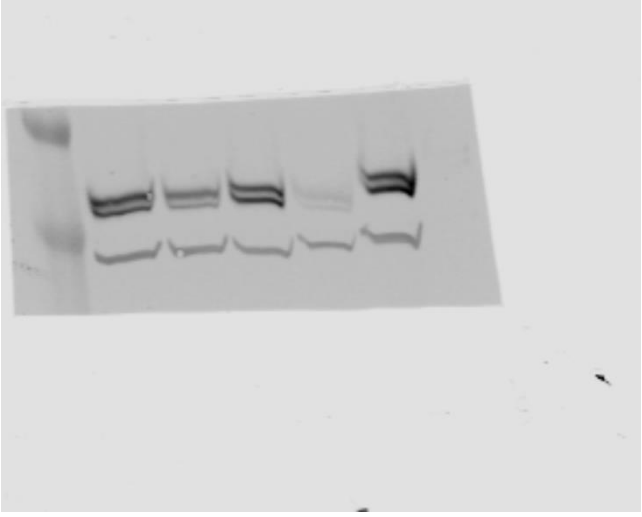

Total AKT	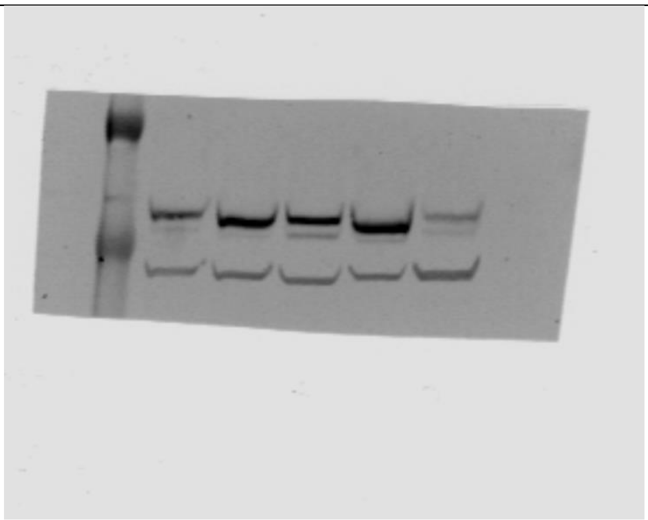
p-ERK	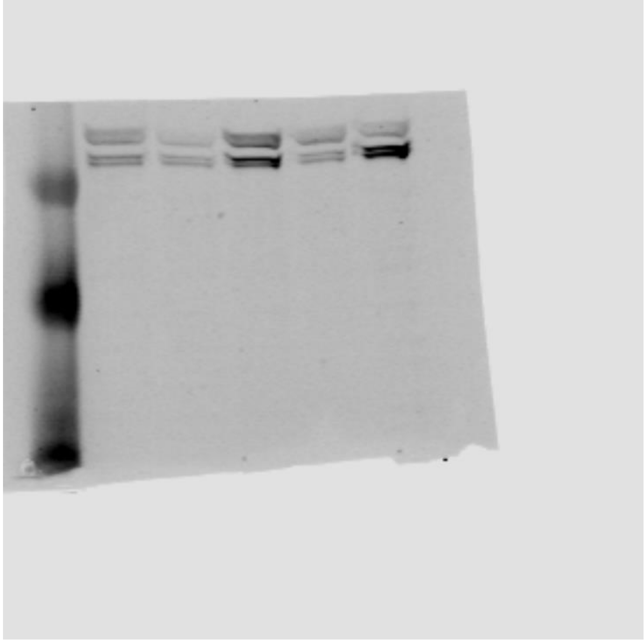
Total ERK	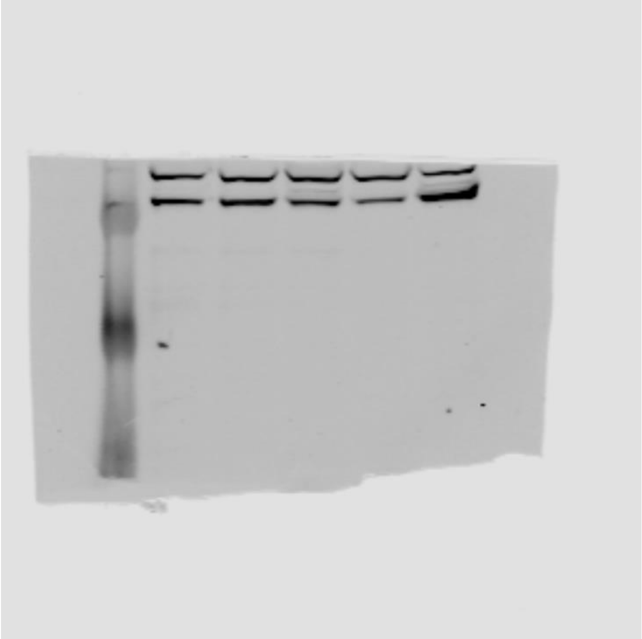

Alpha tubulin	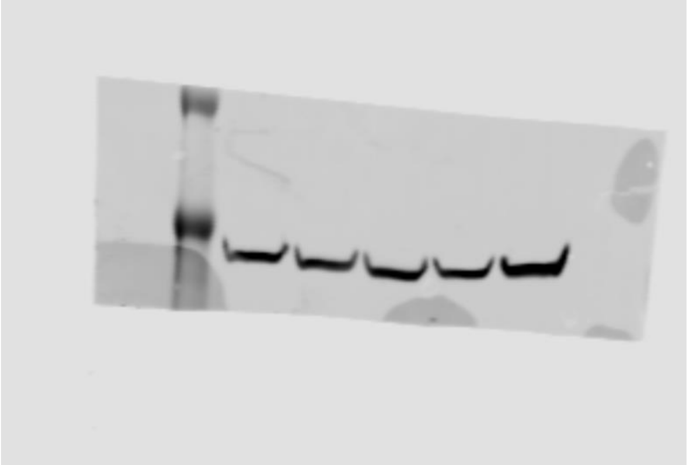

Original blot images For Supplemental Figure S2	
p-EGFR	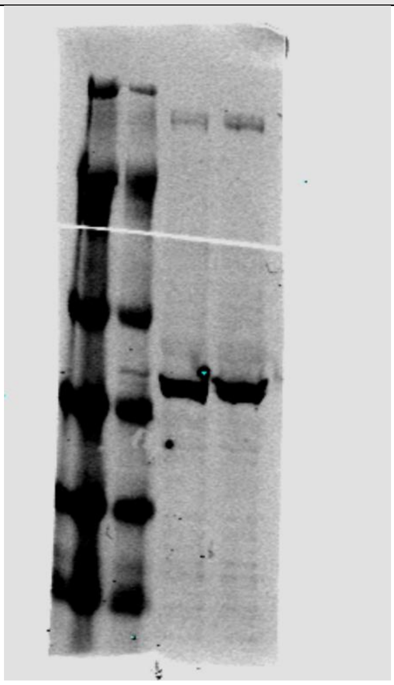
Total EGFR	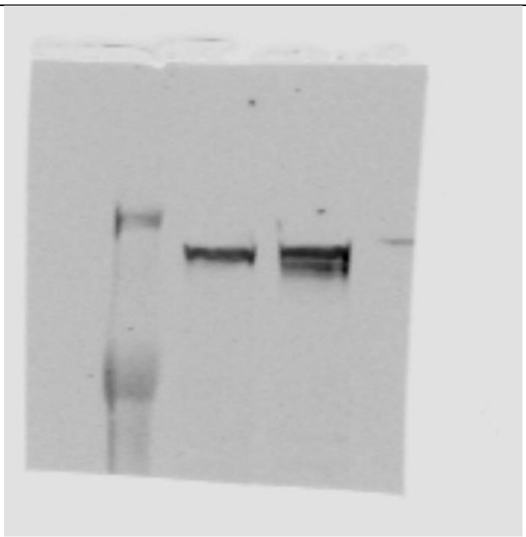

p-HER2	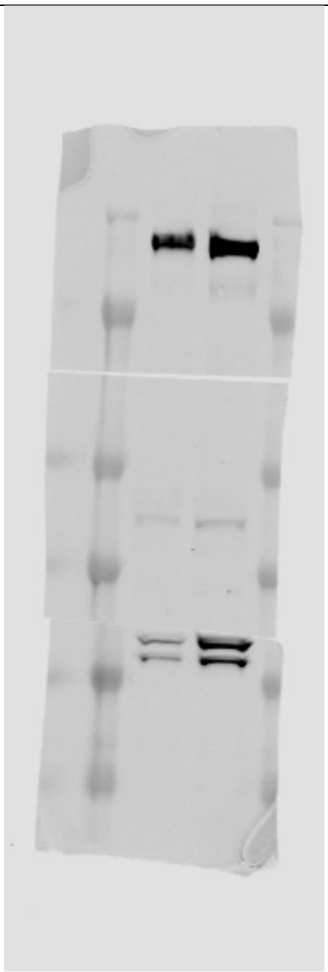
Total HER2	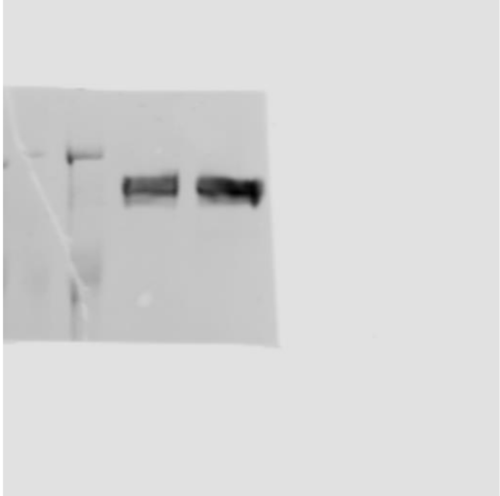

Total ER	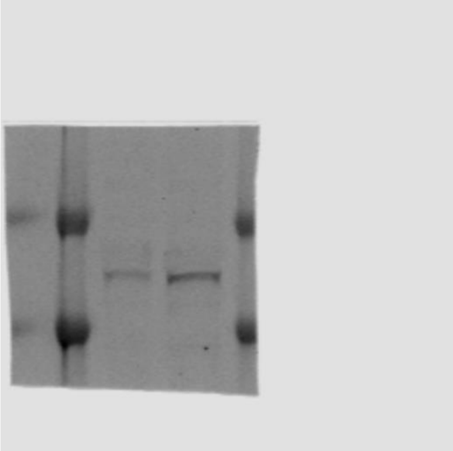
p-AKT	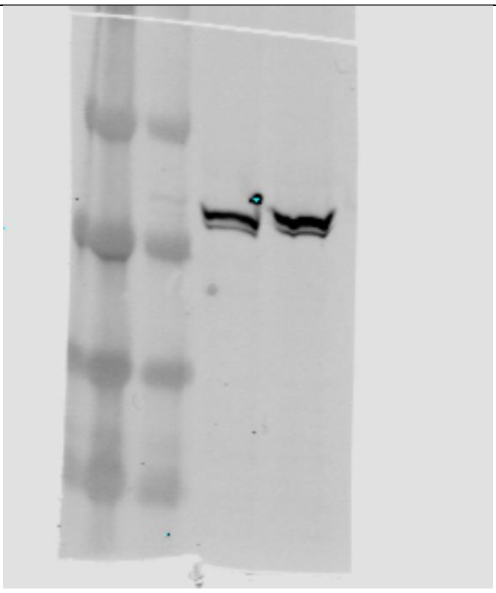
Total AKT	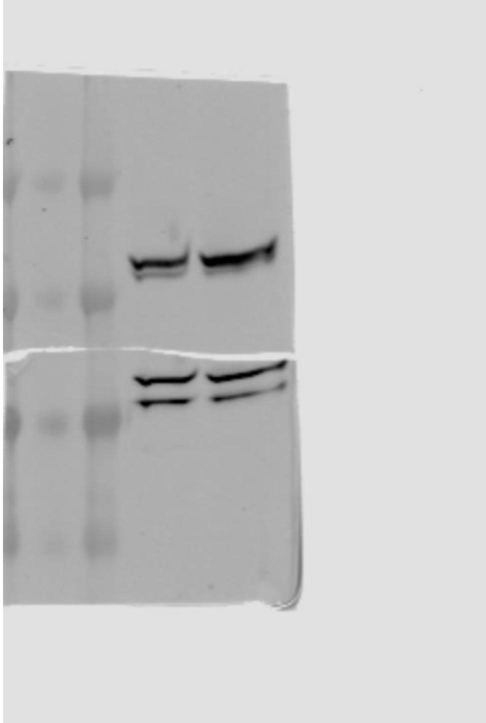

p-ERK	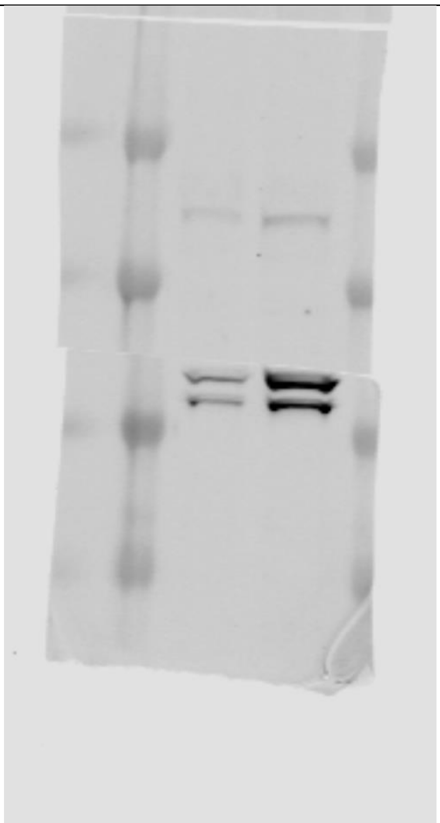
Total ERK	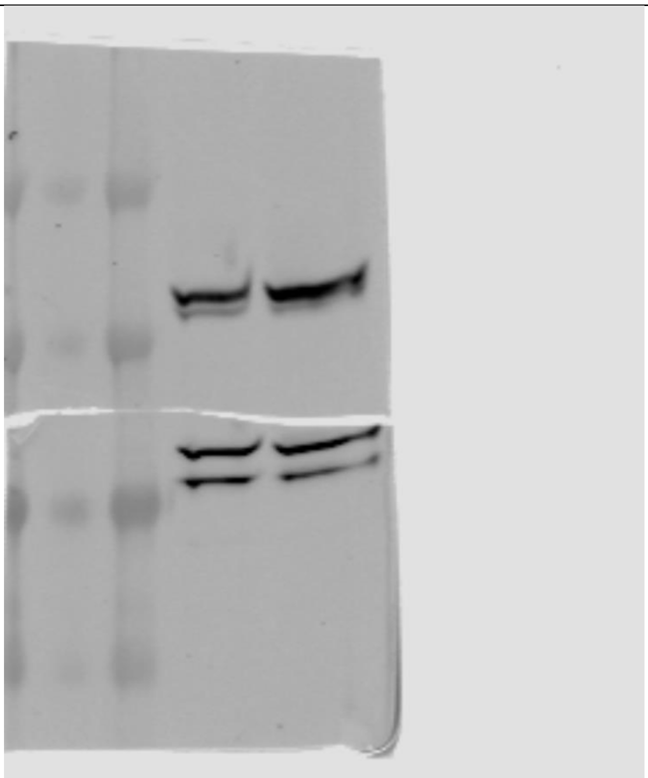

Alpha tubulin	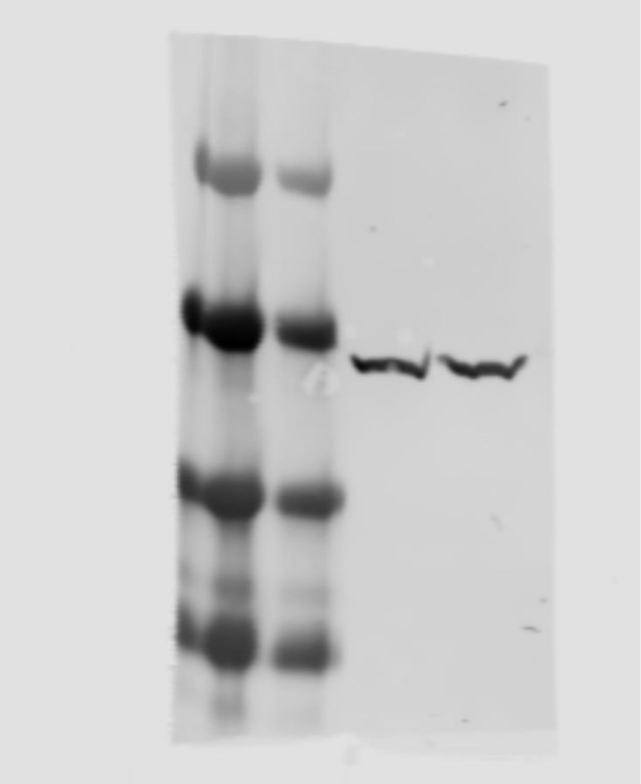
