# Supplementary material for: Pre-Clinical Rationale for Amcenestrant Combinations in HER2+/ER+ Breast Cancer
Source: Int J Mol Sci. 2025 Jan 8;26(2):460. doi: 10.3390/ijms26020460 (PMC11765389; doi:10.3390/ijms26020460)
Supplement: Supplementary file 1 [file ijms-26-00460-s001.zip › 18.12.23 IJMS Amcenestrant supplemental post review final.pdf]

**Supplementary Material**

**Title: Pre-Clinical Rationale for Amcenestrant Combinations in HER2+/ER+ Breast Cancer**

**Author list:** Amira F. Mahdi [1 2]*, Niall Ashfield [1], John Crown [1 3], Denis M. Collins [1]*

**Affiliations:**
1: Cancer Biotherapeutics Research Group, Life Sciences Institute, School of Biotechnology, Dublin City University, Dublin 9, D09 NR58 Dublin, Ireland; niall.ashfield3@mail.dcu.ie (N.A.); john.crown@ccrt.ie (J.C.)
2: imerick Digital Cancer Research Centre, Health Research Institute, School of Medicine, University of Limerick, V94 T9PX Limerick, Ireland
3: DDepartment of Medical Oncology, St. Vincent's University Hospital, Dublin 4, D04 T6F4 Dublin, Ireland

* Correspondence: amira.mahdi@ul.ie (A.F.M.); denis.collins@dcu.ie (D.M.C.)

## Supplementary Tables

**Supplementary Table S1**: Drug concentrations of combinations tested in fixed ratio proliferation assays

| Combination | Amcenestrant (nM) | Neratinib (nM) | Tucatinib and lapatinib (nM) | Tucatinib and Lapatinib in BT474 (nM) | T-DM1 (ng/mL) |
|---|---|---|---|---|---|
| 1 | 5000 | 10 | 500 | 250 | 500 |
| 2 | 1000 | 5 | 250 | 125 | 250 |
| 3 | 200 | 2.5 | 125 | 62.5 | 125 |
| 4 | 40 | 1.25 | 62.5 | 31.25 | 62.5 |
| 5 | 8 | 0.625 | 31.25 | 15.625 | 31.25 |
| 6 | 1.6 | 0.3125 | 15.625 | 7.8125 | 15.625 |

**Supplementary Table S2: Effect of amcenestrant on relative growth of cell lines and subsequent sensitivity classification**

| Cell Lines | % Cell Growth @5 µM Amcenestrant ± SD | Amcenestrant sensitivity |
|---|---|---|
| BT-474 | 85.2 ± 2.2 % | + |
| MDA-MB-361 | 53.3 ± 2.8 % | ++ |
| EFM-192a | 81.8 ± 3.4 % | + |
| BT-474-T | 59.6 ± 7 % | ++ |

**Supplementary Table S3:** Summary of Loewe synergy metrics from Combenefit, regardless of statistical significance. SUM_SYN_ANT_WEIGHTED: the sum of synergy and antagonism weighted according to original dose-response. SYN_MAX: represents the maximum level of synergy observed score for the corresponding drug combination, statistical significance is not taken into account.

| CELL LINE | DRUG | SUM_SYN_ANT_WEIGHT | SYN_MAX |
|---|---|---|---|
| BT-474 | Neratinib | 11.90 | 19.22 |
| | Tucatinib | 16.29 | 16.04 |
| | Lapatinib | 16.82 | 16.03 |
| | T-DM1 | -3.77 | 7.27 |
| MDA-MB-361 | Neratinib | 59.05 | 25.21 |
| | Tucatinib | 64.71 | 23.54 |
| | Lapatinib | 53.56 | 21.78 |
| | T-DM1 | -3.59 | 2.70 |
| EFM-192a | Neratinib | 3.58 | 10.56 |
| | Tucatinib | 2.57 | 11.39 |
| | Lapatinib | 5.02 | 15.95 |
| | T-DM1 | -4.88 | 2.25 |
| BT-474-T | Neratinib | 16.18 | 19.23 |
| | Tucatinib | 16.84 | 27.61 |
| | Lapatinib | 28.04 | 28.19 |
| | T-DM1 | 2.53 | 10.43 |

## Supplementary Figures

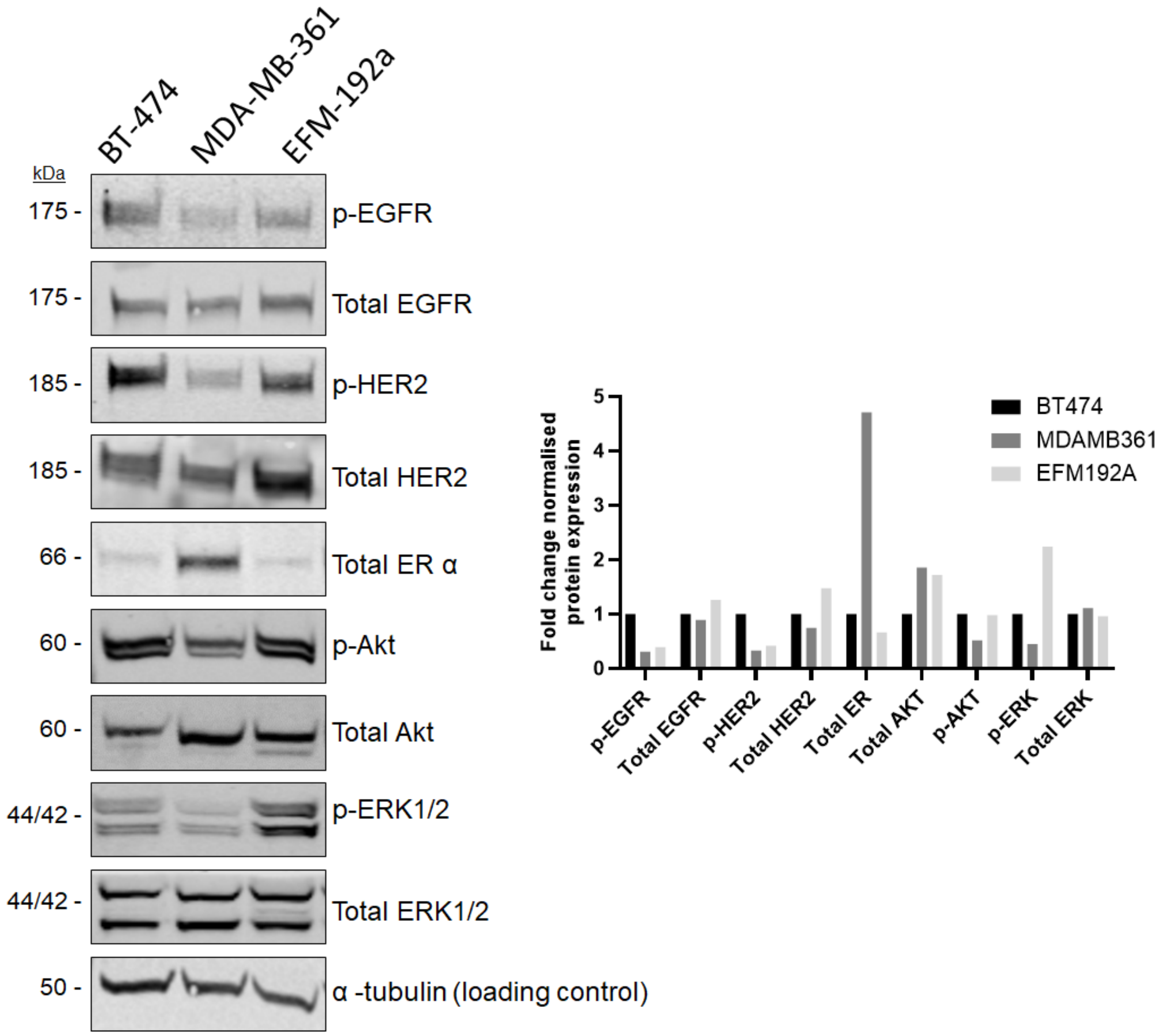


**Supplementary Figure S1:** Verification of the expression of proteins of interest in the chosen cell lines to be used in this study. Western blot analysis of protein expression in BT-474, MDA-MB-361 and EFM-192a cell lines shows expression of both HER2 and ER in these lines, along with a several associated proteins. n=1

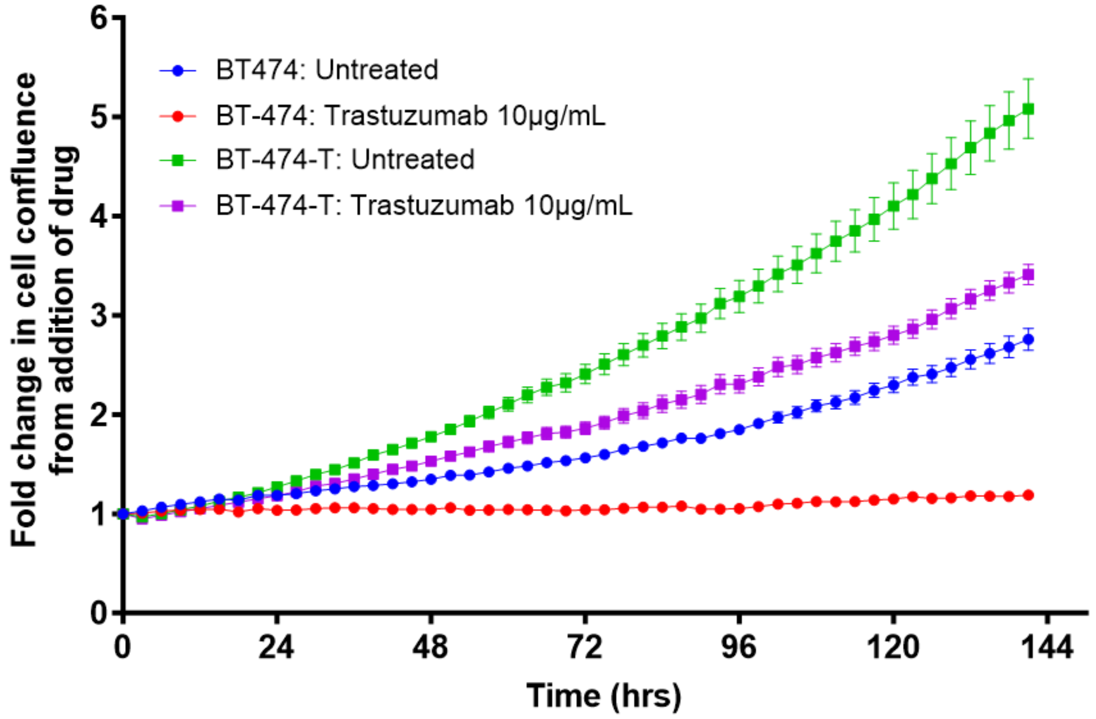


**Supplementary Figure S2:** Growth curve of BT-474 cell lines and BT-474 -T cultured in RPMI or trastuzumab 10 µg/ml. Cells were allowed to adhere for 24 hours before drug treatment. Using the IncuCyte Live Cell Analysis System, images were taken at 10 X magnification every 3 hours, over 6 days to monitor cell confluence. Fold change in cell confluence upon addition of drug was plotted. Points indicate mean ± SEM. n=1

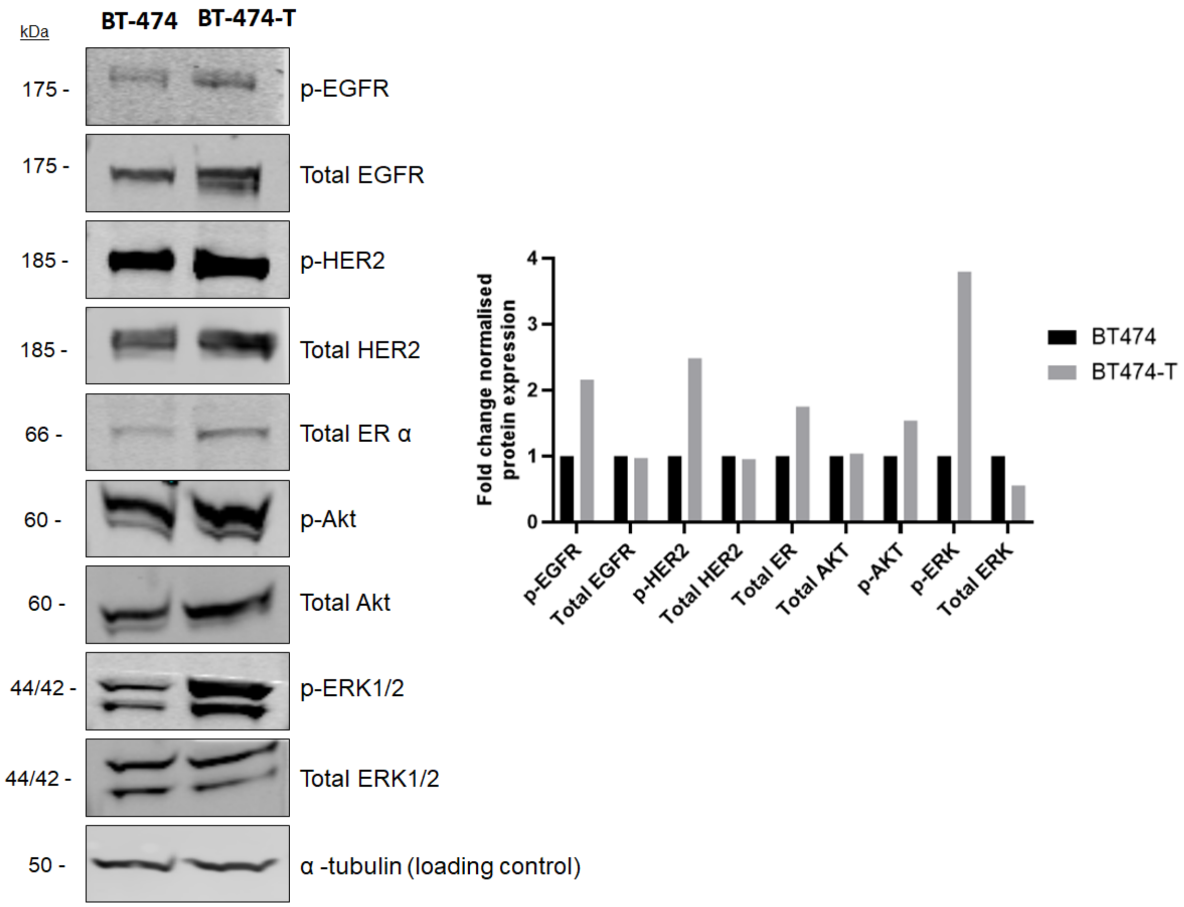


**Supplementary Figure S3:** Verification of the expression of proteins of interest in the chosen cell lines to be used in this study. Western blot analysis of protein expression in BT-474 versus BT-474, shows expression of both HER2 and ER in these lines, along with a several associated proteins. n=1.

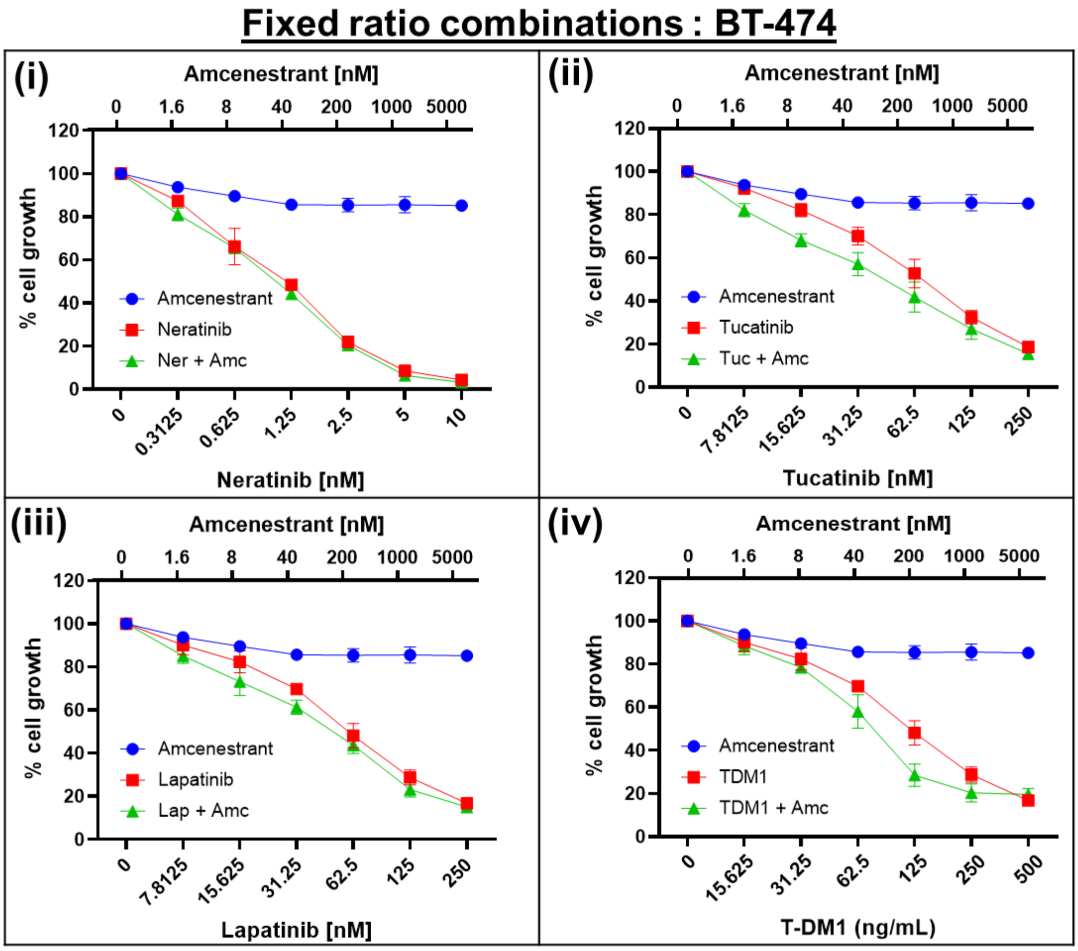


**Supplementary Figure S4:** The effect of HER2 targeted agents and amcenestrant on BT-474 cells following 5 days of treatment, as measured using acid phosphatase proliferation assay. (i)- (iv) BT-474 growth curves following treatment with fixed ratio combinations of HER2 targeted drugs and amcenestrant. n=3

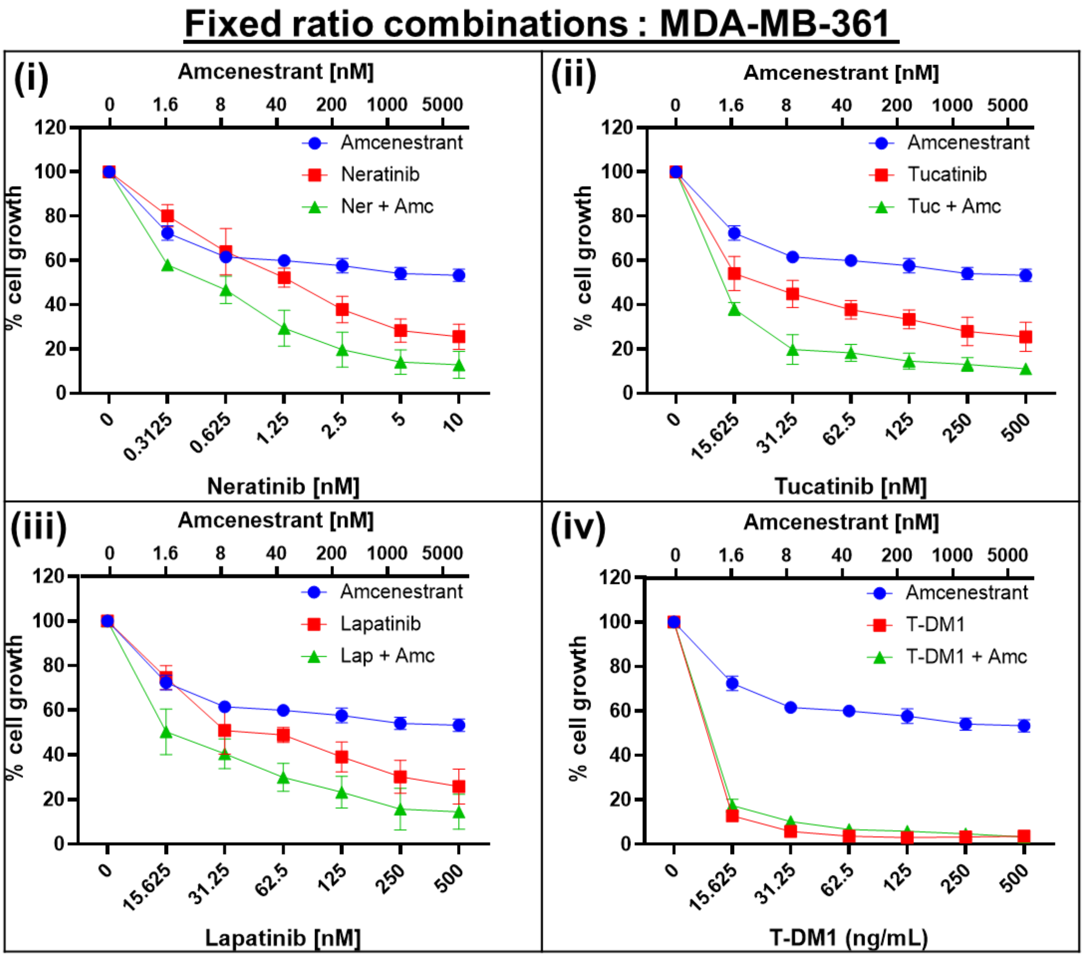


**Supplementary Figure S5:** The effect of HER2 targeted agents and amcenestrant on MDA-MB-361 cells following 5 days of treatment, as measured using acid phosphatase proliferation assay. (i)- (iv) MDA-MB-361 growth curves following treatment with fixed ratio combinations of HER2 targeted drugs and amcenestrant. n=3.

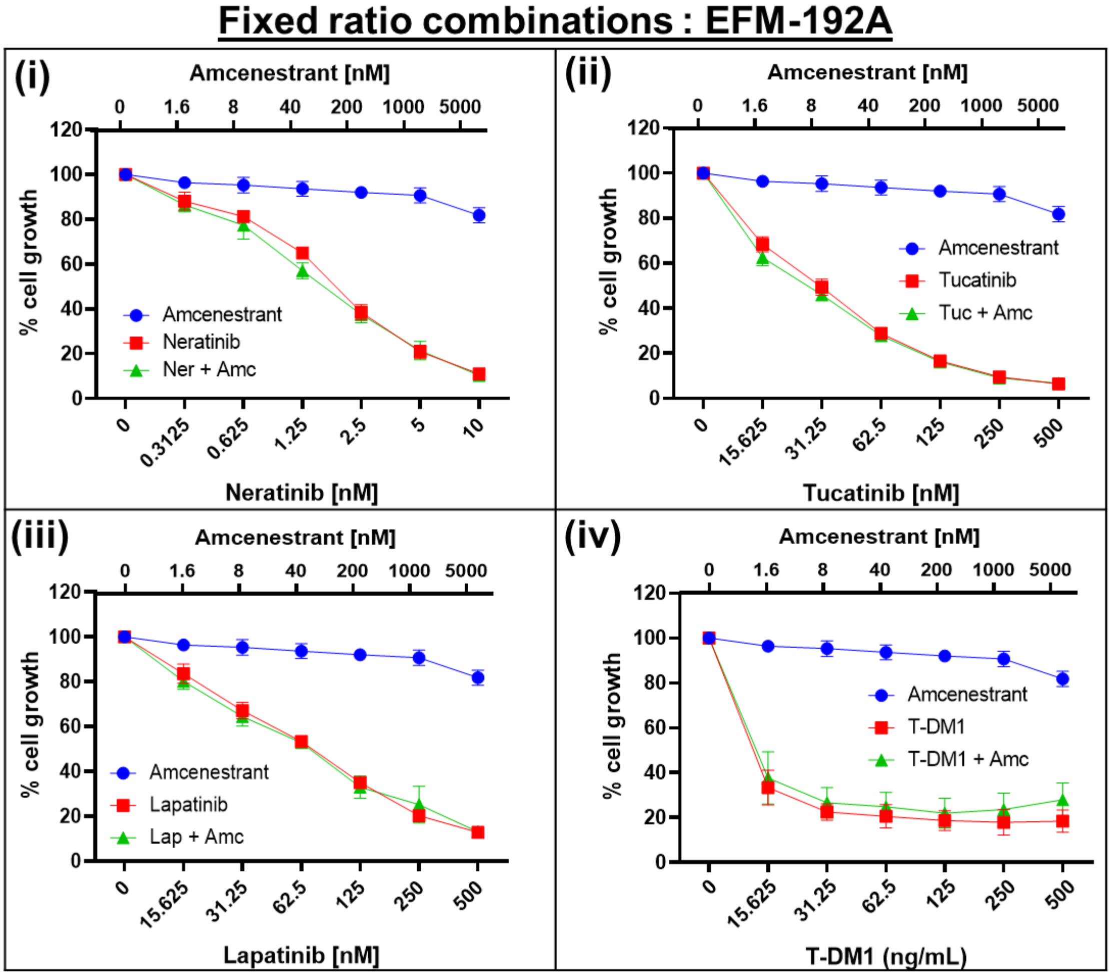


**Supplementary Figure S6:** The effect of HER2 targeted agents and amcenestrant on EFM-192A cells following 5 days of treatment, as measured using acid phosphatase proliferation assay. (i)- (iv) EFM-129A growth curves following treatment with fixed ratio combinations of HER2 targeted drugs and amcenestrant. n=3.

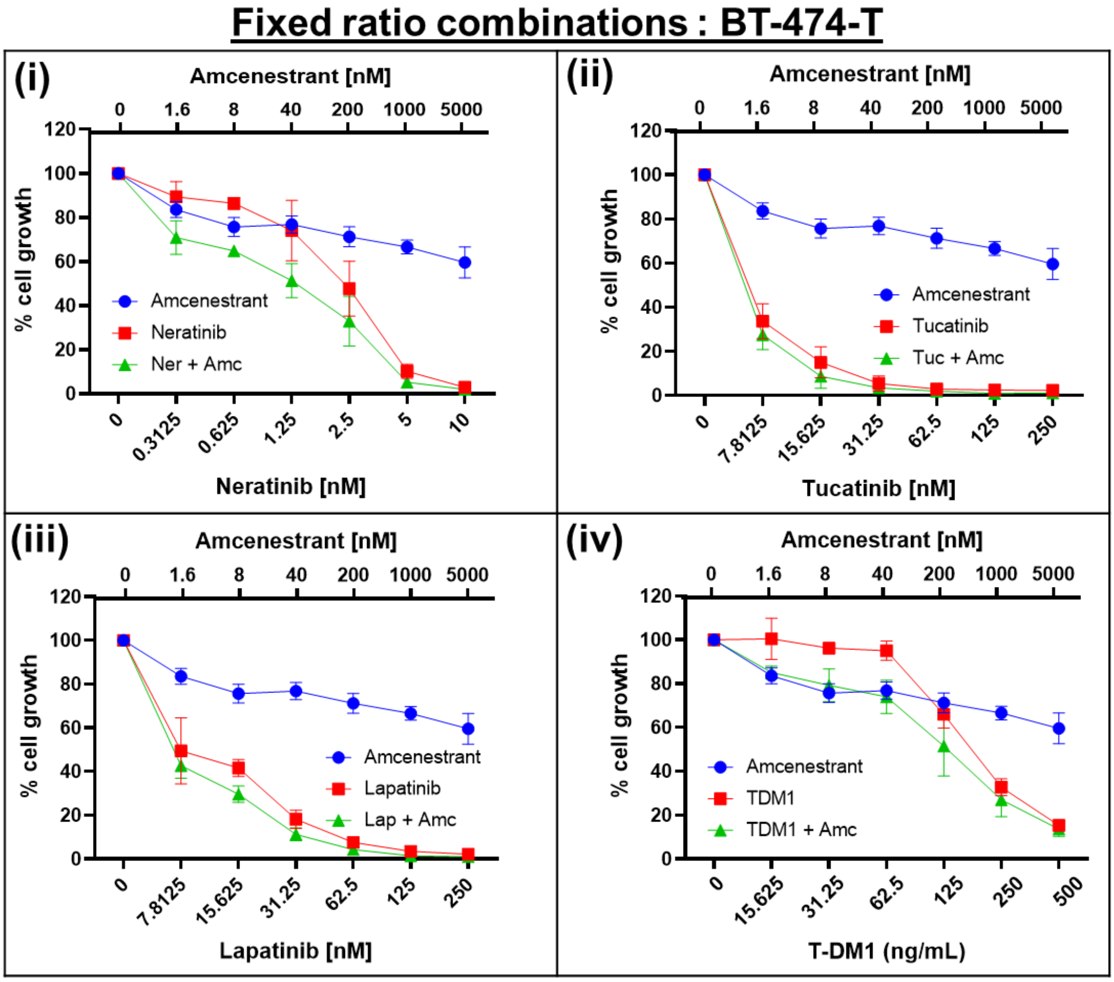


**Supplementary Figure S7:** The effect of HER2 targeted agents and amcenestrant on BT-474-T cells following 5 days of treatment, as measured using acid phosphatase proliferation assay. (i)- (iv) BT-474-T growth curves following treatment with fixed ratio combinations of HER2 targeted drugs and amcenestrant. n=3

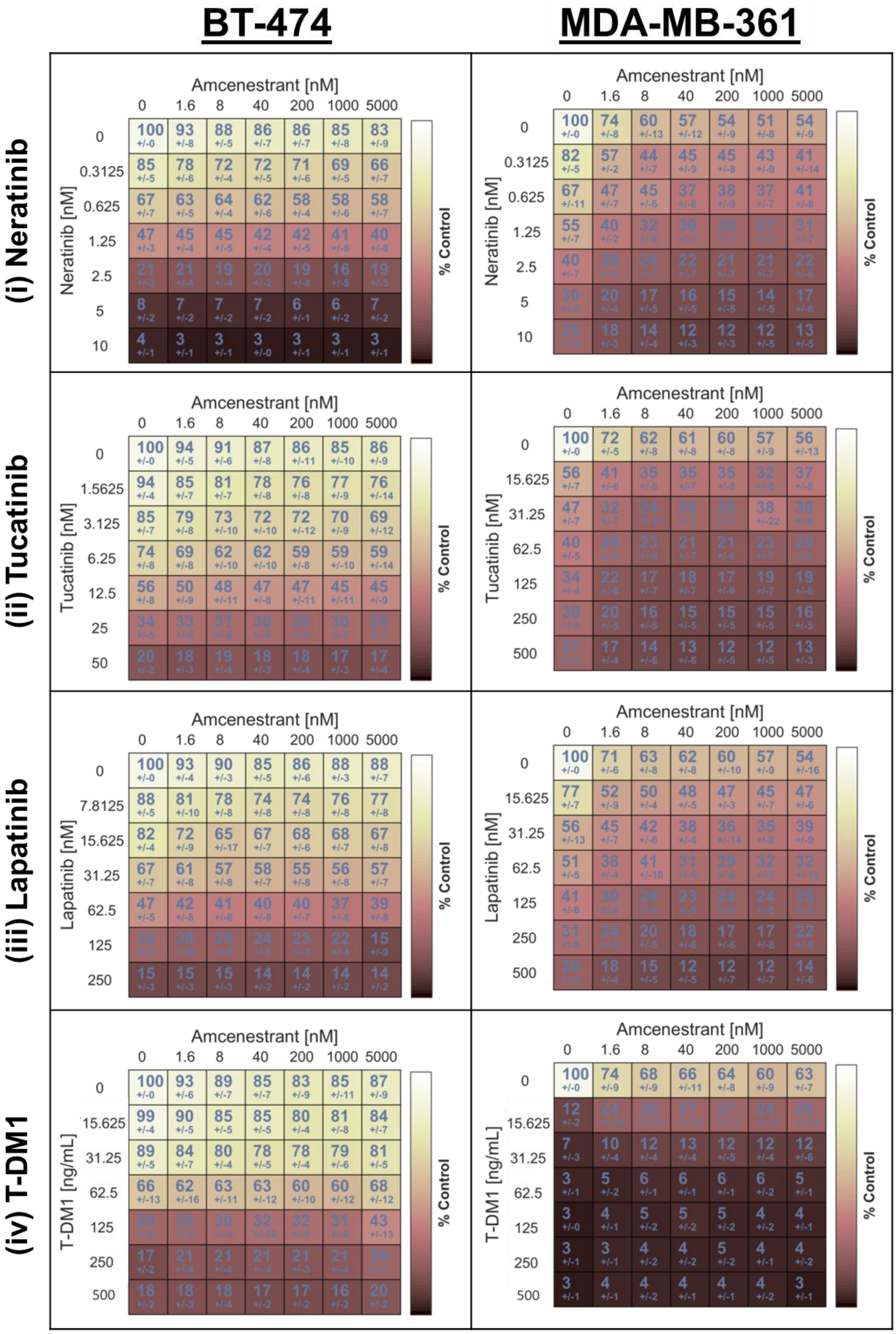
BT-474
MDA-MB-361
(i) Neratinib
(ii) Tucatinib
(iii) Lapatinib
(iv) T-DM1
Amcenestrant [nM]
0 1.6 8 40 200 1000 5000
Neratinib [nM]
0 0.3125 0.625 1.25 2.5 5 10
Tucatinib [nM]
0 1.5625 3.125 6.25 12.5 25 50
0 15.625 31.25 62.5 125 250 500
Lapatinib [nM]
0 7.8125 15.625 31.25 62.5 125 250
0 15.625 31.25 62.5 125 250 500
T-DM1 [ng/mL]
0 15.625 31.25 62.5 125 250 500
% Control

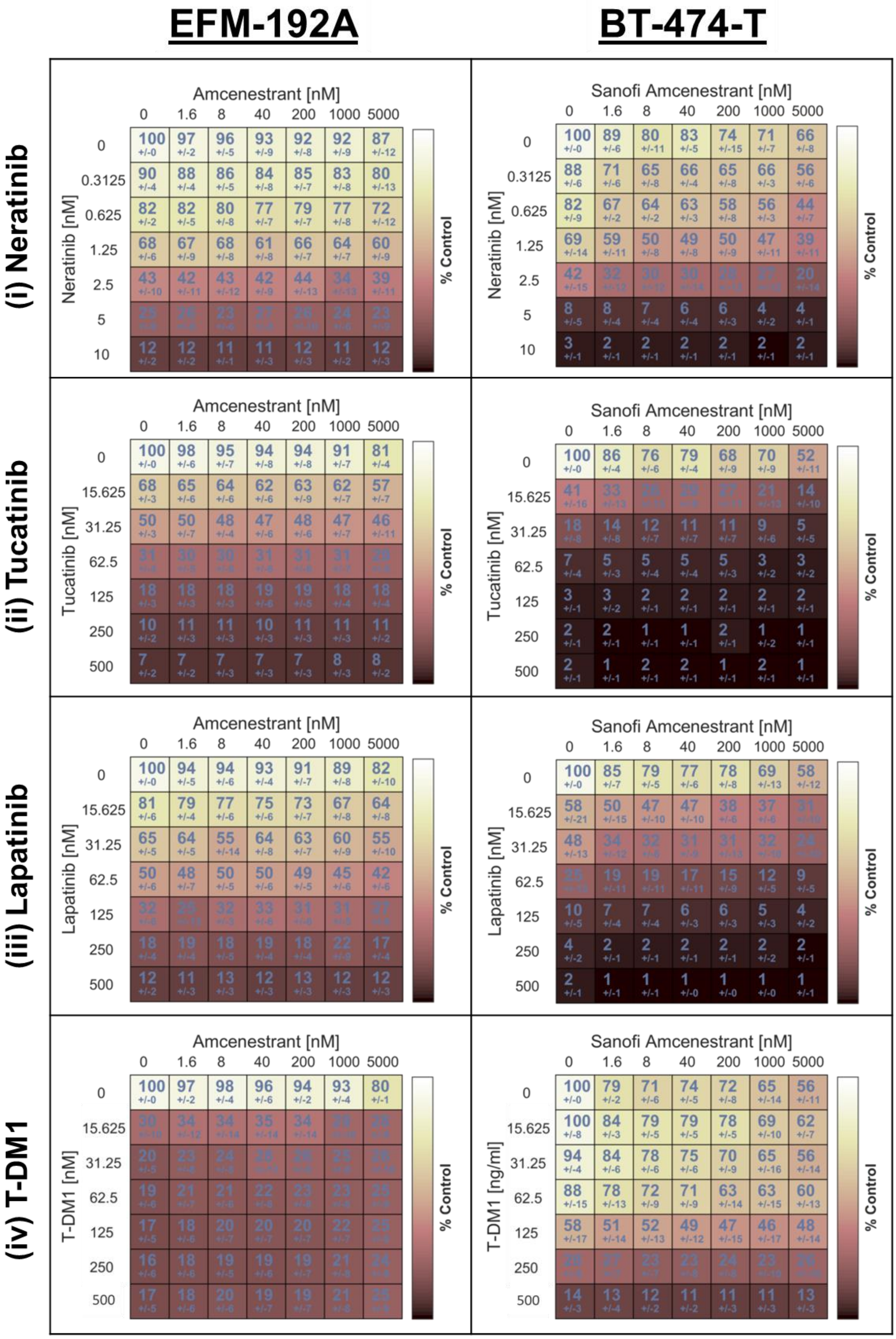
EFM-192A
BT-474-T
(i) Neratinib
(ii) Tucatinib
(iii) Lapatinib
(iv) T-DM1
Amcenestrant [nM]
Sanofi Amcenestrant [nM]
0 1.6 8 40 200 1000 5000
Neratinib [nM]
0 0.3125 0.625 1.25 2.5 5 10
Tucatinib [nM]
Lapatinib [nM]
0 15.625 31.25 62.5 125 250 500
T-DM1 [nM]
T-DM1 [ng/ml]
% Control

**Supplementary Figure S*8:*** Dose-response matrices from combinations of amcenestrant and HER2 targeted therapies, following 5 days of treatment, as measured using acid phosphatase proliferation assay. Values displayed as % growth compared to untreated control. Accompantying analysis of interactions between the combinations of two drugs illusatred in **Figure 3**.  n= 4

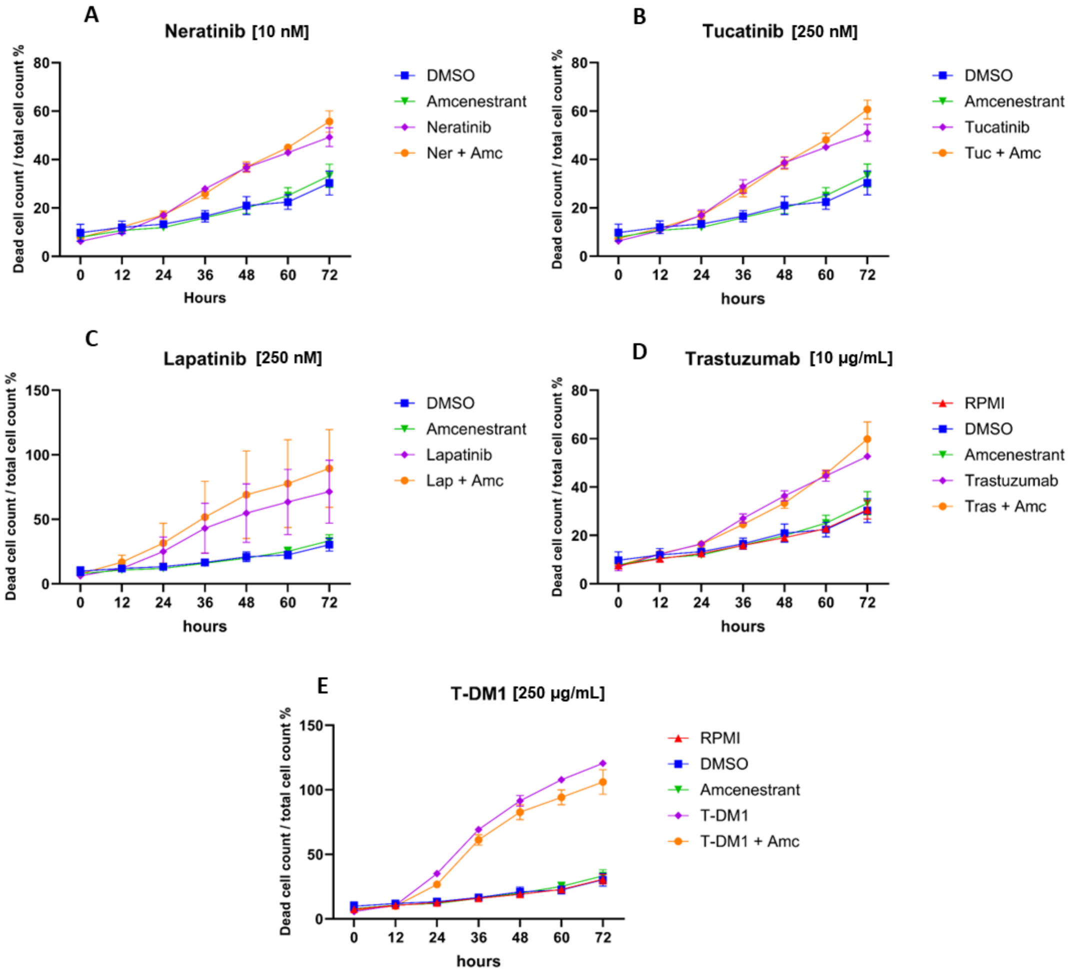


**Supplementary Figure S9: Apoptosis induction in MDA-MB-361 cells after treatment with amcenestrant, HER2 targeted agents and combinations.** Proportion of dead cells to total cell count is graphed every 12 hours, showing increasing levels of apoptosis over time. (A) Neratinib combinations, (B) Tucatinib combinations, (C) Lapatinib combinations, (D) trastuzumab combinations, (E) T-DM1 combinations. Graphs depict mean ± SEM. n=3

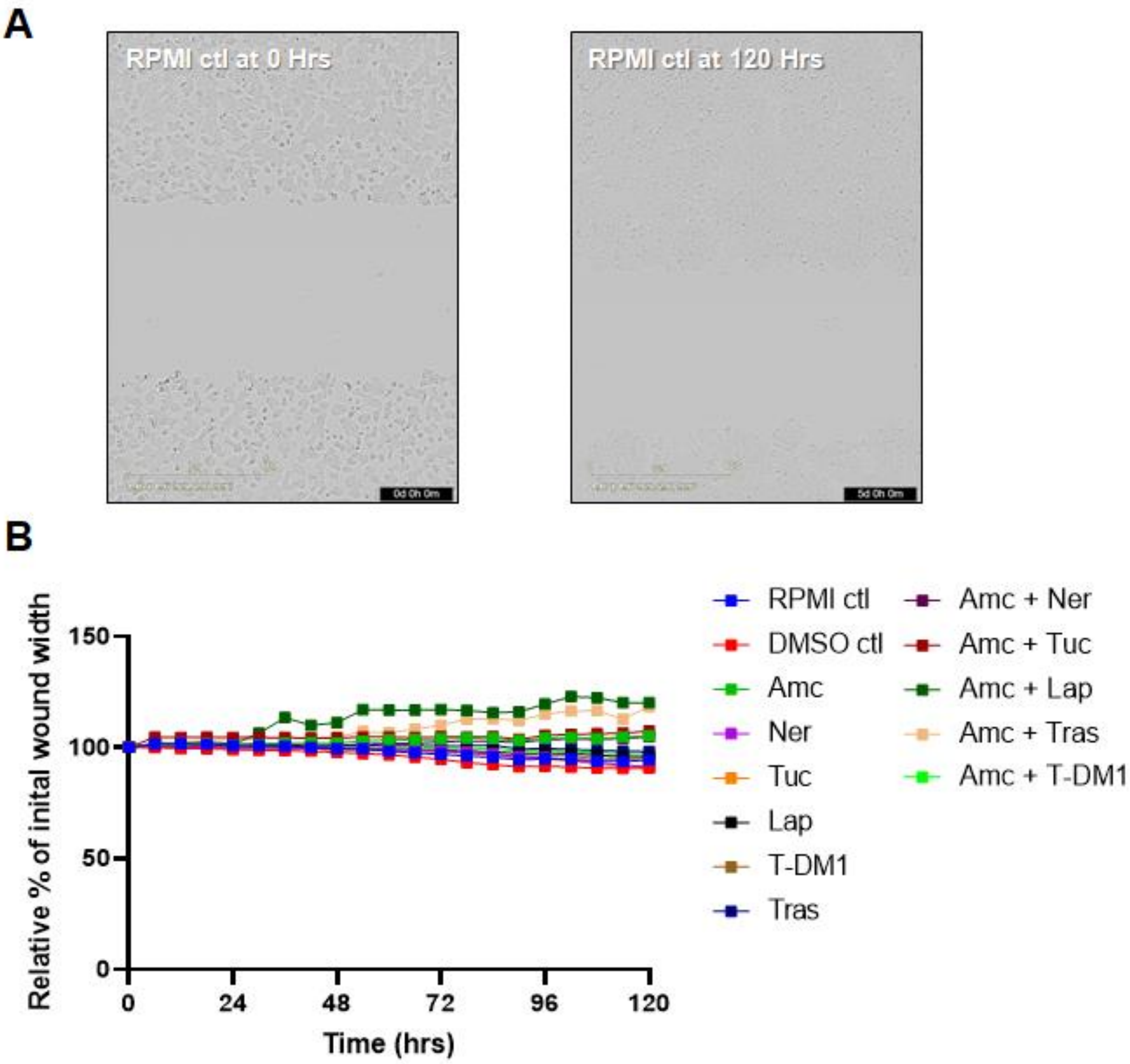


**Supplementary Figure S10: The cell line MDA-MB-361 did not display wound healing/ migratory behaviour following the infliction of a scratch wound, over the course of 120 hrs**. (A) Representative Incucyte microscope images showing RPMI untreated control cells at 0 hrs and 120hr incubation. (B) Wound closure was not observed in any of the tested conditions, expressed as % of the original measured wound, over 120 hrs, data depicts average of 4 images per condition. n=1
